# The WOPR Domain Protein OsaA Orchestrates Development in *Aspergillus nidulans*

**DOI:** 10.1371/journal.pone.0137554

**Published:** 2015-09-11

**Authors:** Fahad Alkahyyat, Min Ni, Sun Chang Kim, Jae-Hyuk Yu

**Affiliations:** 1 Department of Bacteriology, The University of Wisconsin-Madison, Madison, Wisconsin, United States of America; 2 Department of Food Science, The University of Wisconsin-Madison, Madison, Wisconsin, United States of America; 3 Department of Biological Sciences, Korea Advanced Institute of Science and Technology, Dae-Jon, Republic of Korea; University of Nebraska, UNITED STATES

## Abstract

Orchestration of cellular growth and development occurs during the life cycle of *Aspergillus nidulans*. A multi-copy genetic screen intended to unveil novel regulators of development identified the AN6578 locus predicted to encode a protein with the WOPR domain, which is a broadly present fungi-specific DNA-binding motif. Multi-copy of AN6578 disrupted the normal life cycle of the fungus leading to enhanced proliferation of vegetative cells, whereas the deletion resulted in hyper-active sexual fruiting with reduced asexual development (conidiation), thus named as *osaA* (Orchestrator of Sex and Asex). Further genetic studies indicate that OsaA balances development mainly by repressing sexual development downstream of the *velvet* regulator VeA. The absence of *osaA* is sufficient to suppress the *veA1* allele leading to the sporulation levels comparable to *veA* ^+^ wild type (WT). Genome-wide transcriptomic analyses of WT, *veA1*, and Δ*osaA veA1* strains by RNA-Seq further corroborate that OsaA functions in repressing sexual development downstream of VeA. However, OsaA also plays additional roles in controlling development, as the Δ*osaA veA1* mutant exhibits precocious and enhanced formation of Hülle cells compared to WT. The OsaA orthologue of *Aspergillus flavus* is able to complement the *osaA* null phenotype in *A*. *nidulans*, suggesting a conserved role of this group of WOPR domain proteins. In summary, OsaA is an upstream orchestrator of morphological and chemical development in *Aspergillus* that functions downstream of VeA.

## Introduction

Coordination of vegetative growth and reproduction in filamentous fungi requires finely regulated networks of diverse genetic elements, which integrate intrinsic signals with surrounding external cues [1–3]. In the ascomycete *Aspergillus nidulans*, the asexual life cycle starts with the germination of a conidium followed by the formation of an undifferentiated network of interconnected hyphal cells, collectively termed mycelium, which develops according to a functionally coherent plan. Asexual development (conidiation) begins by a series of morphogenetic differentiations results in the formation of conidia (asexual spores) bearing structure known as the conidiophore (reviewed in [4]).

Asexual reproduction is centrally controlled by BrlA, a highly conserved activator of conidiation in the genus *Aspergillus* [5, 6]. Sexual development in *A*. *nidulans*, a morphogenetic counterpart to conidiation, is achieved by the formation of a cleistothecium that bears ascospores (sexual spores). Each cleistothecium is surrounded by a nest-like structure made from thick-walled Hülle cells serving as nursing cells [7]. These two developmental processes are antagonistic, and initiation of one developmental process inhibits the other. Genetic and molecular investigations have revealed several activator of sexual reproduction (reviewed in [8]). To achieve the proper sporulation levels, the fungus utilizes a range of interacting genetic systems [3, 8].

The *velvet* regulators (*veA*, *velB*, *velC* and *vosA*) were found in many fungal species to assume a key role in regulating development; dictate and balance both types of reproductive spores (conidia and ascospores) [9–12]. VeA (interacting with VelB) is required to activate sexual reproduction and indirectly inhibits conidiation [10, 13–15]. In addition, it is involved in activating secondary metabolism (SM) through physically interacting with LaeA, the master activator of SM [10]. The *veA1* knockdown mutation, which lacks nuclear localization signal (NLS), thereby defective in the translocation to the nucleus, causes significantly reduced activity, resulting in highly reduced sexual fruiting with enhanced conidiation, i.e., the *velvet* phenotype [14, 16].

The WOPR domain proteins are a newly defined family of regulators that started gaining attention for the past decade. WOPRs are a fungi-specific family of transcriptional factors that are involved in multiple biological processes in various fungi [17, 18]. The name WOPR is derived from the best-studied member Wor1, and its closely related members Pac2 and Ryp1 [18]. WOPR proteins regulate morphological transitions and pathogenesis in many fungi: e.g., Ryp1 in *Histoplasma capsulatum* [19] and Wor1 in *Candida albicans* [20] and Liv3 in *Cryptococcus neoformans* [21]. They were also found to play crucial roles in regulating sporulation in a number of fungi, including *Fusarium* spp. [22, 23]. The WOPR protein Ryp1 functions along with the *velvet* homologs (Ryp2 and Ryp3) in controlling hypha-to-yeast developmental switch in *H*. *capsulatum* [19, 24].

In an attempt to further understand the genetic networks underlying developmental regulation in *A*. *nidulans*, we carried out a gain-of-function multi-copy screen. OsaA with a predicted WOPR domain was identified as a potential regulator of development. Further genetic and genomic studies have revealed that OsaA plays a pivotal role in orchestrating asexual and sexual development primarily by down-regulating sexual fruiting downstream of VeA in *A*. *nidulans*. Our studies uncover a new genetic interaction between the WOPR protein OsaA and the key *velvet* regulator VeA, and they together function in coordinating developmental life cycle in *A*. *nidulans*. A new genetic model depicting the roles of VeA and OsaA is presented.

## Material and Methods

### Fungal strains and culture conditions

The *Aspergillus* strains used in this study are listed in Table 1. Standard culture and genetics techniques were used [25]. Strains were grown on minimal solid or liquid medium (simplified as MM) with appropriate supplements as previously described [26] at 37°C unless otherwise indicated. Induction of asexual development or sexual development was done as described previously [27, 28].

**Table 1:** *Aspergillus* strains used in this study.

Strain	Genotype	Source/Reference
*A*. *nidulans* ^a^		
FGSC4	*veA* ^*+*^ WT	FGSC^b^
FGSC26	*biA1* WT	FGSC
FGSC237	*pabaA1*, *yA2; trpC801*	FGSC
PW1	*biA1; argB2; methG1*	P. Weglenski
RRAW16	*pyrG89*, *yA2; veA* ^*+*^	R. A. Wilson and N. P. Keller
RYG1.9	*pabaA1*, *yA2; argB2*, *ΔfluG*::*trpC* ^*+*^	Guan and Yu, unpublished
TNI3.1	*argB2*; *pyroA4*; Δ*vosA*::*argB* ^*+*^	This study
RNIW5	*pyrG89; pyroA4*	This study
RNI11.2, 3, 4^C^	*biA1; ΔosaA*::*argB* ^*+*^	This study
RNI15.1, 2, 3C	*biA1; ΔosaA*::*argB* ^*+*^ *; veA* ^*+*^	This study
TNI18.1, 2, 3, 4, 5^C^	*pabaA1; yA2; gpdA(p)*::*osaA*::*trpC* ^*+*^	This study
TFA4.1, 2^C^	*biA1; ΔosaA*::*argB* ^*+*^ *; biA* ^*+*^ *; osaA* ^*+*^	This study
TFA5.1, 2^C^	*biA1; ΔosaA*::*argB* ^*+*^ *; biA* ^*+*^ *; AflwprA* ^*+*^	This study
*A*. *flavus*		
NRRL 3375	Wild type	[29]

^a^All *A*. *nidulans* strains carry the *veA1* mutation, unless mentioned as *veA* ^+^.

^b^FGSC: Fungal Genetics Stock Center

^c^Multiple isogenic strains.

### Gain of function genetic screen

The recipient strain RNIW5 (*pyrG89 pyroA4 veA* ^*+*^) was transformed with the pRG3-AMA1-*Not*I WT library [30], which confers multi-copy presence of a given gene. The transformants showing fluffy phenotypes were isolated. Genomic DNA was isolated from these fluffy transformants, and was transformed into *Escherichia coli* to recover the plasmids. The rescued plasmids were introduced back into the recipient strain to check whether they could still cause fluffy phenotype. By direct sequencing of the insert ends of the interested plasmids with the primer set OMN33 and OMN35 (all primers listed in S2 Table), and followed by genome search [31] identified several potential repressors of development. Four such developmentally altered transformants identified, including AN6578, which is renamed as *osaA*. To examine the positions of *osaA’s* introns, RT-PCR, followed by sequencing analyses, was carried out. Note that the gene structure of *osaA* is different from the predicted structure of AN6578.3 from the Broad Institute [31].

### Construction of fungal strains

*A*. *nidulans osaA* deletion mutant (TNI3.1) was generated by transforming PW1 with the *osaA* deletion cassette containing *argB* ^+^ as the selective marker. The cassette was constructed by employing Double-Joint PCR [32]. RNI11.1 (Δ*osaA veA1*) was isolated from the cross between TNI2.1 and TNI3.1. RNI11.2, 3, 4 (Δ*osaA veA1*) were isolated from the cross between TNI3.1 and RYG1.9. RNI15.1, 2, 3 (Δ*osaA veA* ^*+*^) were isolated from the cross between RNI11.1 and RRAW16. TFA4.1,2 (Δ*osaA veA1 osaA(p)*::*osaA* ^*+*^) were generated by transforming RNI11.3 (*biA1*) with the *osaA* gene PCR fragment including 1.6 kb from each of 5’ and 3’ region. TFA5.1,2 (Δ*osaA AflwprA(p)*::*AflwprA* ^*+*^ *veA1*) were generated by transforming RNI11.3 (*biA1*) with the *AflwprA* gene PCR fragment including 1.6 kb from each of 5’ and 3’ region from *A*. *flavus* NRRL 3375 strain [29].

### Nucleic acid isolation and manipulation

Genomic DNA and total RNA isolation and Northern blot analyses were carried out as previously described [27, 33]. The DNA probes were prepared by PCR-amplification of a coding region of individual genes with appropriate oligonucleotide pairs using FGSC4 genomic DNA as template (S2 Table).

### Microscopy

The colony photographs were taken using a Sony DSC-F828 digital camera. Photomicrographs were taken using a Zeiss M^2^ BIO microscope equipped with AxioCam and AxioVision digital imaging software (Zeiss).

### Sample preparation for mRNA sequencing

Three biological replicates were analyzed for each strain. All strains were cultured in agitating liquid-submerged medium (vegetative growth) for 18 h, and then shifted to an air-exposed medium to induce development. All samples were collected at time point 12 h following developmental induction, total RNA was extracted, and submitted to ProteinCT Biotechnologies (Madison, WI) for library preparation and sequencing.

### Library preparation and sequencing

Strand specific library was prepared from total RNA using Illumina TruSeq Strand specific RNA sample preparation system. Briefly, mRNA was extracted from total RNA using polyA selection, followed by RNA fragmentation. Strand specific library was constructed by first-strand cDNA synthesis using random primers, followed by sample cleanup and the second-strand synthesis using DNA Polymerase I and RNase H. A single 'A' base was added to the cDNA fragments followed by ligation of the adapters. Final cDNA library was obtained by further purification and enrichment with PCR, and the quality was checked using Bioanalyzer 2100. The library was sequenced (PE100bp) using the Illumina HiSeq2500, with final of over 10 million high quality reads per sample. RNA-Seq full workflow integrated service was provided by ProteinCT Biotechnologies (Madison, WI). The scattered plot in Supplementary S1 Fig shows a high correlation levels among triplicates, indicated by the correlation coefficient *R* (*R*-Δ*osaA veA1* > 0.97, *R*-*veA1* WT > 0.97 and *R*-*veA* ^*+*^ WT > 0.97), all with the p-value less than 0.01.

### Data QC and analysis

The fastQC program was used to verify raw data quality of the Illumina reads. The *A*. *nidulans* FGSC4 genome and gene annotations (A_nidulans_FGSC_A4_version_s10-m03-r08_features.gff) were downloaded from AspGD [34, 35] and used for mapping. The raw sequence reads were mapped to the genome using Subjunc aligner from Subread [36], with majority of the reads (80–90% for all samples) aligned to the genome. The alignment bam files were compared against the gene annotation GFF file, and raw counts for each gene were generated using the featureCounts tool from Subread, with ~70–85% of reads assigned to genes overall. The raw counts data were normalized using voom method from the R Limma package [37], then used for differential expression analysis. For transcripts assembly, Cufflinks program [38–41] was used to assemble transcripts from RNA-Seq reads for each sample, and cuffmerge command from cufflinks was used to merge the transcripts from all samples into a single set of genes and transcripts. The gene annotation from AspGD was provided as reference in the process and included in the final assembly.

## Results

### Multi-copy screen identifies AN6578 as a regulator of development

**Fig 1:**
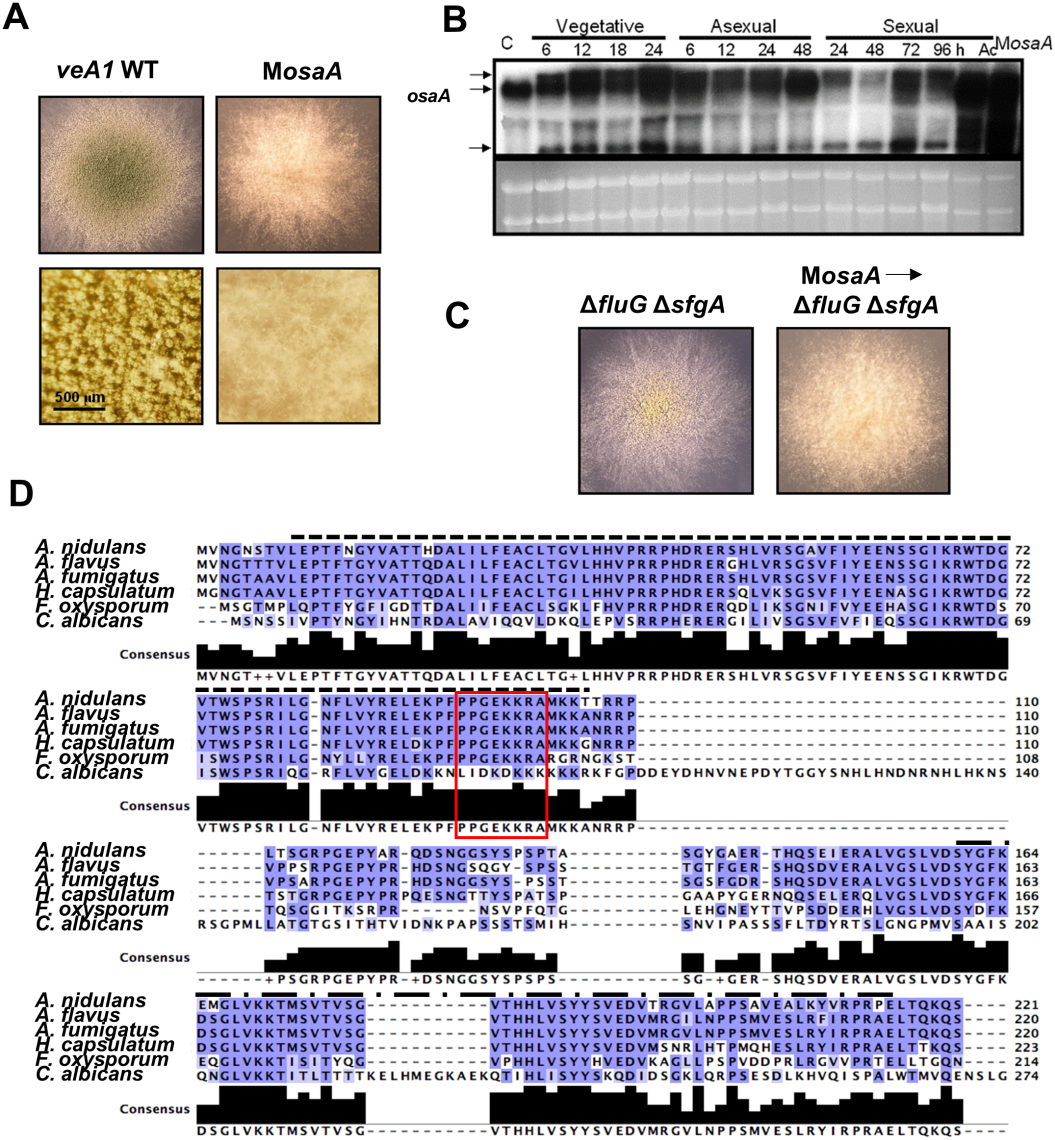
Summary of OsaA. (A) Colony photographs of WT and the multi-copy *osaA* strains (upper panel) and close-up views (lower panel). (B) Northern blot showing the *osaA* transcripts [~3.2kb, ~2.8kb and ~1kb] levels throughout the life cycle. C: conidia, Ac: ascospores, M*osaA*: multi-copy *osaA*. (C) Colonies of Δ*fluG* Δ*sfgA* and multi-copy *osaA* (M*osaA*) in Δ*fluG* Δ*sfgA* strains grown on solid MM at 37°C for 3 d along with the close-up views (lower panel). (D) Alignment of the WOPR proteins in *Aspergillus* and other WOPR members. *A*. *nidulans* OsaA (AN6578), *A*. *flavus* (AFL2T_08419), *A*. *fumigatus* (Afu5g12960), (ABX74945.1), *F*. *oxysporum* Sge1 (AGA55574.1), *F*. *graminearum* (I1S5P3) and *C*. *albicans* Wor1 (Q5AP80). The dashed line indicates the conserved region 1, and the dash-dot line indicates the conserved region 2. The red box indicates the NLS sequence.

To investigate the mechanisms governing fungal development, a gain-of-function (multi-copy) screen was carried out as described in (11). We envisioned that such regulators are unlikely to be defined via chemical mutagenesis, which leads to mostly loss of function mutations. As described (11), after screening more than 56,000 transformants, seven colonies exhibiting no or reduced developmental phenotypes were isolated, and four of them contained AN6578 in the multi-copy plasmid pRG3-AMA1 [30]. Re-introduction of the multi-copy plasmid containing the AN6578 locus alone resulted in a near complete loss of development with fluffy phenotype (Fig 1A). Further studies showed the AN6578 gene was required for balancing asexual and sexual development, thus named as ***osaA*** (**o**rchestrator of **s**exual and **a**sexual development). Constitutive expression of *osaA* by *gpdA*(p)::*osaA* delayed development about 24h (data not shown).

Northern blot and sequence analyses of the RT-PCR products suggested that the *osaA* gene consists of three overlapping transcripts (~3.2 kb, ~2.8 kb and ~1 kb; S4 Fig). Only the 2.8 kb transcript is present in conidia (C), while all three transcripts are detectable during vegetative growth, asexual and sexual development with the highest accumulation levels in sexual spores (ascospores; Ac in Fig 1B), suggesting OsaA might play a role during the life cycle of the fungus. The two long transcripts (3.2 kb and 2.8 kb) contain the identical 1,422 nt ORF, which is translated into a 474 amino acids (aa) polypeptide. The small transcript is derived from an alternative splicing of the 1,422 transcript, and the 43^rd^ to 860^th^ nt segment of the OsaA long ORF is removed in the final exon, leading to a 149 aa-length protein. All these findings imply that the activity of *osaA* might be regulated at the transcription level.

### OsaA functions independently to the FluG mediated conidiation pathway

FluG is a key upstream activator of asexual development. The deletion of *fluG* results in complete blockage of conidiation [42]. FluG activates conidiation via removing repressive effects imposed by SfgA [43]. As the data indicate that OsaA may acts as a repressor of conidiation, we asked whether OsaA functions in the FluG—| SfgA governed conidiation pathway. We generated and examined the phenotypes of the Δ*osaA* Δ*fluG* mutant, which were similar to as those of Δ*fluG* (data not shown). Moreover, the Δ*osaA alcA*(p)::*sfgA* (overexpression of *sfgA*) double mutant showed phenotype similar to that of the *alcA*(p)::*sfgA* mutant (data not shown), indicating OsaA may function upstream of, or independent to, the FluG—| SfgA pathway. Importantly, multi-copy of *osaA* (M*osaA*) in the Δ*fluG* Δ*sfgA* background resulted in a developmental phenotype in-between the two mutants, (Fig 1C), indicating that OsaA does not function in upstream of FluG—|SfgA pathway.

### *OsaA* encodes a WOPR domain protein

Through BLAST search [31] we found that the *osaA* gene is predicted to encode a WOPR domain protein at the N-terminus (Fig 1D) with a similarity in amino acid sequence to Wor1 N-terminus in *C*. *albicans* [18, 20, 44]. The predicted OsaA polypeptide (473 aa) is highly conserved in all *Aspergillus* species. The N-terminus, in particular, is conserved in almost all filamentous fungi and it harbors the WOPR domain (Fig 1D). WOPR was shown to have sequence-specific DNA binding property; therefore, it represents a novel DNA-binding protein superfamily [18, 24, 44]. The predicted OsaA protein contains the two-conserved WOPR subdomains, Conserved Region 1 (CR1) and Conserved Region 2 (CR2), separated by a characteristic less conserved linker [17, 18]. In addition, OsaA contains the nuclear localization signal (NLS: 95-PGEKKRA-102) within the WOPR domain as found in two other WOPR proteins, HcRyp1 [19, 24] and FoSge1 [23]. Like many fungi [17, 18], *A*. *nidulans* has two predicted WOPR proteins, OsaA and WprB.

### OsaA represses sexual development downstream of VeA

**Fig 2:**
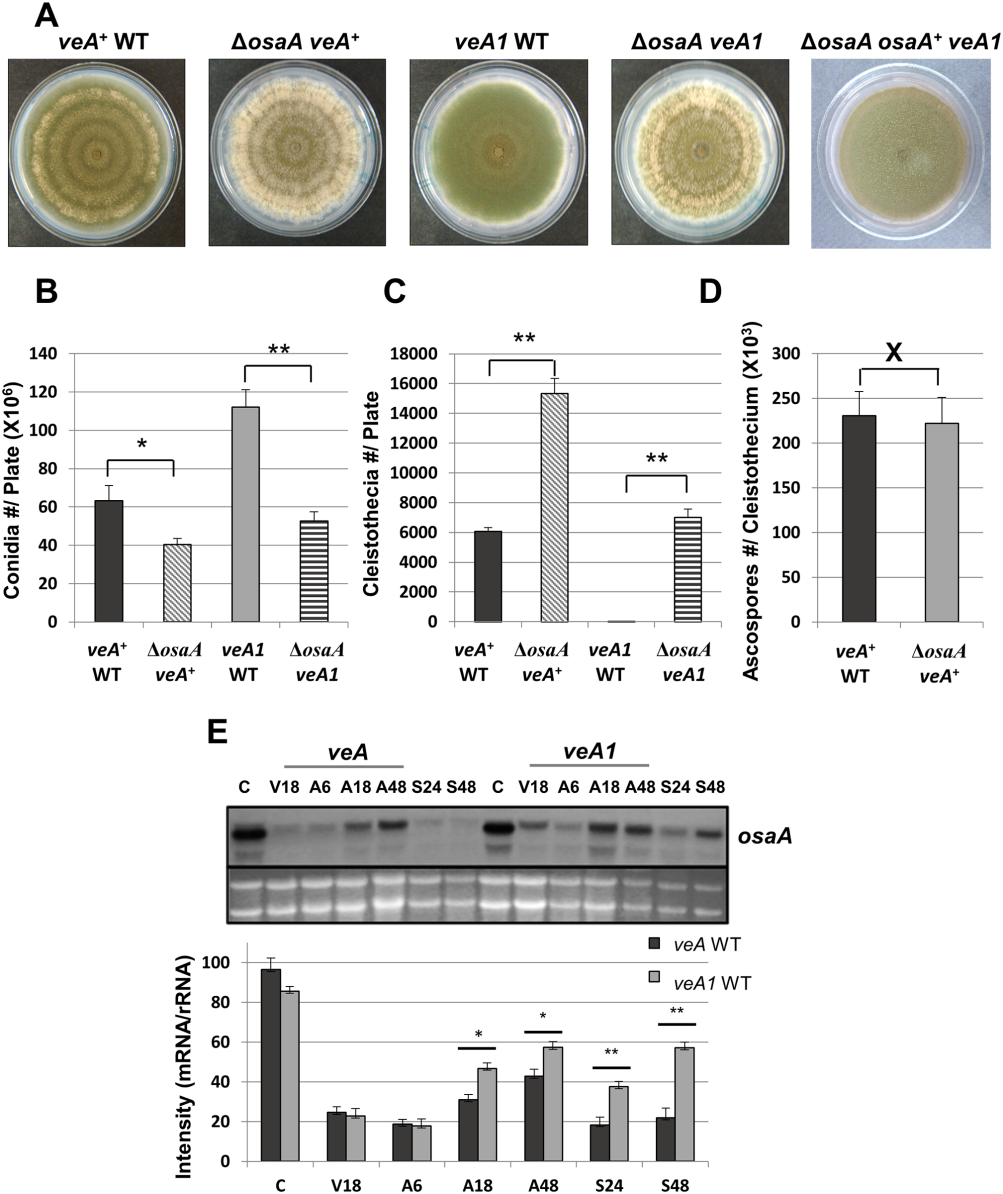
OsaA balances sporulation levels. (A) Colony photographs of WT (*veA* ^***+***^ and *veA1*), *osaA* mutants (Δ*osaA veA* ^***+***^ and Δ*osaA veA1*), and *osaA* complementation (Δ*osaA osaA veA1*) strains. (B) A histogram depicting the number of conidia per plate. (C) A histogram depicting the number cleistothecia per plate. (D) A histogram depicting the number (10^3^) ascospores per cleistothecium. In B, C and D error bars represent the standard deviation among three or more replicates; * represents p-value < 0.05; ** represents p-value < 0.01; X represents p-value > 0.5. (E) Northern blot showing *osaA* mRNA accumulation levels in *veA* ^*+*^ and *veA1* WT strains (Upper). C: conidia; V18: vegetative 18 h; A6, 18, 48: asexual induction 6, 18, 24 h; S24, 48: sexual induction 24, 48 h. Band intensity estimation in reference to the rRNA levels using imageJ software [46] (Lower); * represents p-value < 0.05; ** represents p-value < 0.01.

To begin to understand the function of OsaA, through employing Double-Joint PCR [32], we first generated the *osaA* deletion, in a *veA* ^*+*^ strain mutant, with *argB* ^+^ as a selective marker (RRAW16, Table 1). VeA is required for sexual development, and the *veA1* allele lacking the NLS is partially active thereby causing significantly suppressed sexual reproduction [14–16, 45]. Thus, to properly address the function of OsaA in developmental balancing, we further isolated Δ*osaA veA1* (RNI11) and Δ*osaA veA* ^*+*^ (RNI15) recombinant strains through subsequent genetic crosses. As shown in Fig 2A, both Δ*osaA* mutant colonies displayed clearly altered developmental phenotypes with significantly higher levels of cleistothecia and much reduced levels of conidiation in comparison to their corresponding WTs. Quantitative analyses of conidiation and sexual fruiting revealed the absence of *osaA* resulted in 1.6 (*veA* ^+^) and 2.1 (*veA1*) fold reduction in conidia formation (Fig 2B), while causing 2.5 (*veA* ^+^) and ~ 7,000 (*veA1*) fold enhanced formation of cleistothecia (Fig 2C). It is important to note that the data showing the enhanced production of cleistothecia by Δ*osaA* are obtained from the solid air-exposed cultures grown for 6 days under the light. Light inhibits sexual fruiting in *A*. *nidulans* and almost completely abolishes cleistothecia formation in *veA1* strains. These indicate that OsaA is a key repressor of sexual development and the absence of *osaA* can compensate the inhibitory effects on sexual development by light [1, 3].

On the other hand, the loss of *osaA* did not affect the spore viability or number. The number of ascospores per cleistothecium was similar between the Δ*osaA* and WT strains. However, factoring in the large number of cleistothecia produced by the Δ*osaA* mutant, the total number of ascospores per colony (each point inoculated and cultured for 6 days) is higher in Δ*osaA* compared to WT. In addition, germination and survival rates for both spore types were identical between Δ*osaA* and WT. Collectively, the primary role of OsaA is to repress the onset of sexual development.

Importantly, our data indicate that the loss of *osaA* is sufficient to suppress the *veA1* mutation in development. As shown in Fig 2B and 2C, the Δ*osaA veA1* mutant and *veA* ^*+*^ WT showed similar levels of conidiation and sexual fruiting, indicating that *osaA* is epistatic to *veA1* and *osaA* likely functions downstream of *veA* in regulating sporulation. However, the absence of *osaA* was not sufficient to restore the mycotoxin sterigmatocystin production levels to that of *veA* ^+^ WT (data now shown), suggesting that OsaA is likely not involved in the VelB-VeA-LaeA controlled pathways for certain secondary metabolites [9, 10, 47]. Northern blot showed that levels of *osaA* mRNA are higher in *veA1* WT in contrast to those in *veA* ^+^ WT, especially during sexual development initiation phase, 24 h and 48 h (Fig 2E). Taken together, we propose that OsaA is an upstream repressor of sexual development and VeA removes the repressive effects imposed by OsaA on sexual fruiting.

### Distinct role of OsaA in repressing Hülle cell formation

**Fig 3:**
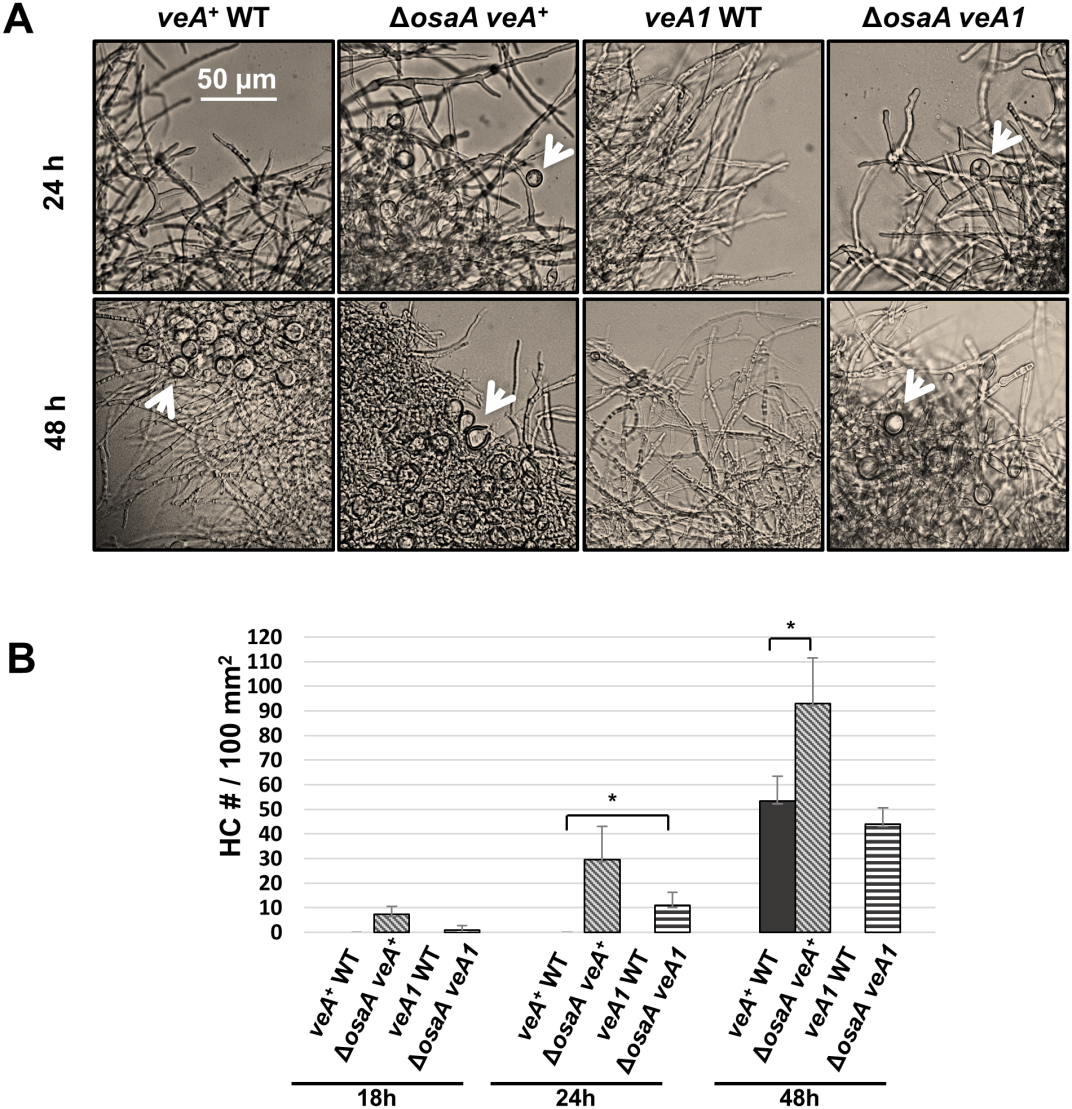
OsaA represses Hülle cell formation. (A) Photomicrographs of WT (FGSC4 and FGSC26) and *osaA* mutant (Δ*osaA veA* ^***+***^ and Δ*osaA veA1*) strains grown in liquid-submerged culture up to 48 h. White arrows indicate Hülle cells. (B) Quantitative analysis of HCs within 100 mm^2^ area. Error bars represent the standard deviation among three replicates; * represents a p-value < 0.05.

Hülle cell (HC) formation is coordinated with the cleistothecium developmental stages, yet, it is proposed to be under the regulation of a separate genetic pathway [7, 15, 48, 49]. Similar to the OsaA’s role in repressing sexual development on air-exposed solid medium, we found that OsaA represses the formation of HC in liquid submerged culture. The Δ*osaA veA* ^*+*^ mutant shows precocious production of HC (as early as 18 h) compared to *veA* ^*+*^ WT strain showing HC production at 48 h (Fig 3A and 3B). At 48 h Δ*osaA veA* ^*+*^ strain produces almost double the number of HC in comparison to *veA* ^*+*^ WT. Surprisingly, even the Δ*osaA veA1* mutant started to produce HC at 24 h, earlier than *veA* ^*+*^ WT strain. These suggest that OsaA is a dual function upstream regulator of sexual development controlling both pathways governing cleistothecia and HC formation.

### Genome-wide suppression of *veA1* by Δ*osaA*

Balancing asexual and sexual development involves the coordinated regulations of thousands of genes associated with cellular, morphological, and metabolic processes. The fact that the deletion of *osaA* was sufficient to suppress the developmental bias toward conidiation caused by the *veA1* mutation suggests that genome-wide expression is likely restored to *veA* ^+^ WT level. To test this hypothesis, we sequenced total RNA populations in Δ*osaA veA1*, *veA1* WT, and *veA* ^*+*^ WT strains at 12 h post asexual-developmental induction.

Fragments per kilobase of exon per million fragments mapped (FPKM) obtained for Δ*osaA veA1*, *veA1* WT, and *veA* ^*+*^ WT strains were mapped for 10,536, 10,428 and 10,514 genes, respectively, representing 96.3%, 95.3% and 96% of the total of 10,943 genes predicted by the AspGD (S2 Fig) [34, 35]. FPKM mapped to the *osaA* locus in the three tested strains confirmed that no *osaA* transcript is detectable in Δ*osaA veA1* strain and higher levels of *osaA* mRNA are present in *veA1* compared to *veA* ^*+*^ WT (S3 Fig). From the data, any differentially expressed genes with a false discovery rate (FDR) greater than 0.05 was considered insignificant. Gene expression changes with slight fold change (FC), i.e., -1< log_2_FC <1, were not included in our analysis, unless stated otherwise.

**Fig 4:**
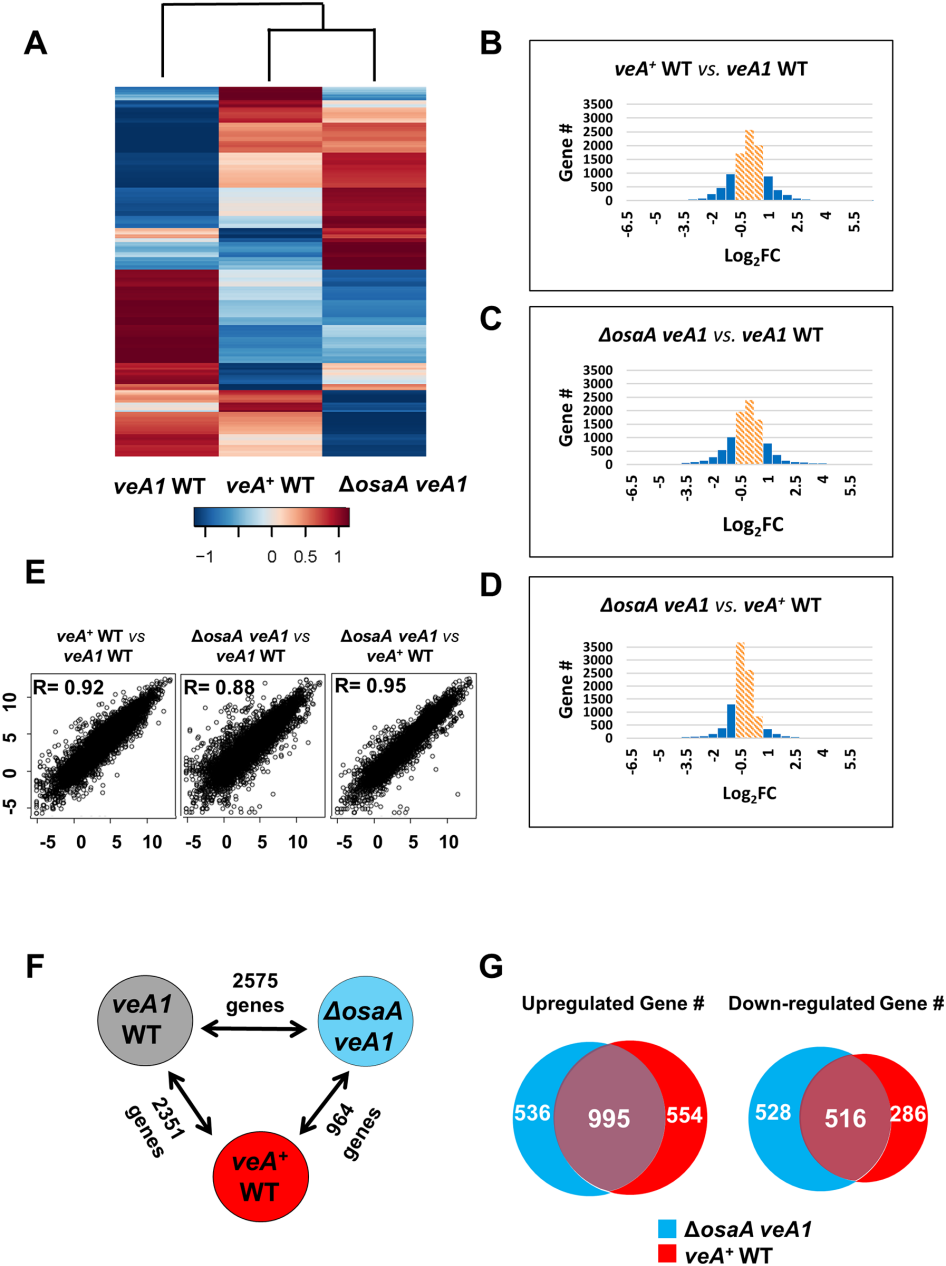
Genome-wide expression correlations among *veA1* WT, *veA* ^*+*^ WT and *ΔosaA veA1*. The included genes all have an FDR value less than 0.05 and fall in the -1<log_2_FC<1 fragment count range, unless indicated otherwise. (A) Heat map illustration of expression level changes among *veA1* WT, *veA* ^*+*^ WT and *ΔosaA veA1* strains. RPKM values were used for hierarchical clustering of genes. Scale bar shows the RPKM values (Z-score). In all analyses *veA1* WT was used as a reference. (B, C, and D) Histograms showing general transcriptomic results of three analytical sets, *veA* ^*+*^ WT vs *veA1* WT, *ΔosaA veA1* vs *veA1* WT and *ΔosaA veA1* vs *veA* ^*+*^ WT, respectively. Columns in orange diagonal lines fall in the 1>log_2_FC>-1 fragment count range with low differential expression values. (E) Linear fitted models showing the correlation between three analytical sets, *veA* ^*+*^ WT vs *veA1* WT, *ΔosaA veA1* vs *veA1* WT and *ΔosaA veA1* vs *veA* ^*+*^ WT. The correlation coefficient *R* is indicated within each model. (F) The numbers of genes showing differential expression between two strains. (G) Venn diagrams showing up- and down-regulated genes in *ΔosaA veA1* and *veA* ^*+*^ WT relative to *veA1* WT. The numbers of common and unique genes are indicated.

As shown in Fig 4A, the hierarchical clustering heat map based on genome-wide expression analysis shows that the transcriptome of Δ*osaA veA1* and *veA* ^*+*^ WT strains are clustered in one group separated from that of the *veA1* WT strain, suggesting that Δ*osaA veA1* shows higher similarity of gene expression pattern to *veA* ^*+*^ WT strain than to *veA1* WT. The degree of differential expression between Δ*osaA veA1* and *veA* ^*+*^ WT is lower than those between *veA1* WT and Δ*osaA veA1*, or *veA1* WT and *veA* ^*+*^ WT strains (Fig 4B, 4C and 4D), further indicating that the distance between the gene expression profiles of Δ*osaA veA1* and *veA* ^*+*^ WT strain is closer than those of the two strains and *veA1* WT. Likewise, we examined the correlation among the overall transcriptomic profiles of the three strains via multidimensional scaling analysis (S5 Fig), and found that Δ*osaA veA1* triplicates fall closer to *veA* ^*+*^ WT than to *veA1* WT. Moreover, by using the gene expression profiles of Δ*osaA veA1*, *veA* ^*+*^ WT and *veA1* WT, we calculated the correlation between differential expression values comparing *veA* ^*+*^ WT to *veA1* WT, Δ*osaA veA1* to *veA1* WT, and Δ*osaA veA1* to *veA* ^*+*^ WT. The obtained correlations have *R-*value equals to 0.92, 0.88, and 0.95, respectively (Fig 4E). These results further indicate that Δ*osaA veA1* and *veA* ^*+*^ WT gene expression patterns are closer to each other than to *veA1* WT, suggesting that suppression of *veA1* by Δ*osaA* is likely due to restored genome-wide expression.

To further analyze differentially expressed genes, pair-wise comparisons were carried out (Fig 4F and 4G). First, the *veA1* and *veA* ^*+*^ data set comparison identified 2,351 differentially expressed genes, 1,549 up-regulated and 802 down-regulated genes. Second, the *veA1* and Δ*osaA veA1* data set comparison revealed 2,575 differentially expressed genes, 1,531 up-regulated and 1,044 down-regulated genes. The obtained high numbers of differentially expressed genes further indicate that both VeA and OsaA act upstream in regulating gene expression, affecting a wide range of downstream genes. Third, the *veA* ^*+*^ and Δ*osaA veA1* data set comparison identified 964 differentially expressed genes, 480 up-regulated and 484 down-regulated genes (Fig 4F). Among the differentially regulated genes, 995 and 516 genes were up-regulated and down-regulated in both Δ*osaA veA1* and *veA* ^*+*^ WT, respectively (Fig 4G). Many in the top 20 differentially expressed gene lists (Tables 2, 3, 4 and 5) are common between Δ*osaA veA1* and *veA* ^*+*^ WT. In a simplified interpretation, common genes are regulated by the *veA* **—|** *osaA* pathway, whereas other genes are probably regulated by either VeA or OsaA independently.

**Table 2:** Top 20 up-regulated genes in Δ*osaA veA1* relative to *veA1* WT.

ID	Log_2_FC	Notes
**AN11049**	11.566	NmrA-like domain containing protein; predicted secondary metabolism gene cluster member
**AN8105**	10.679	Putative NRPS-like enzyme; predicted secondary metabolism gene cluster member
AN1627	10.503	Protein of unknown function
**AN10044**	8.893	*mdpK*
**AN3568**	8.820	Protein of unknown function
**AN0146**	8.631	*mdpC*
**AN5430**	8.612	Protein of unknown function
AN0223	8.491	Has domain(s) with predicted sequence-specific DNA binding RNA polymerase II transcription factor activity, zinc ion binding activity, role in regulation of transcription, DNA-dependent and nucleus localization
**AN11037**	8.407	Predicted transmembrane transporter; predicted secondary metabolism gene cluster member
AN0147	8.381	*mdpD*
**AN6784**	8.319	*xptA*
AN8106	8.058	Predicted dioxygenase; role in secondary metabolite biosynthesis; predicted secondary metabolism gene cluster member
**AN0149**	7.688	*mdpF*
**AN1242**	7.606	Putative NRPS involved in nidulanin A biosynthesis; predicted backbone enzyme of the secondary metabolite gene cluster
**AN10023**	7.483	*mdpL*
**AN6314**	7.399	Has domain(s) with predicted nucleotide binding, oxidoreductase activity and role in metabolic process
AN2595	7.358	Protein of unknown function
AN5539	7.254	Has domain(s) with predicted methyltransferase activity and role in methylation
AN6446	7.080	*cicD*
AN1309	7.077	Has domain(s) with predicted GTP binding, GTPase activity

*Genes that are up-regulated in both Δ*osaA veA1* and *veA* ^+^ WT are in bold.

**Table 3:** Top 20 up-regulated genes in *veA* ^+^ WT relative to *veA1* WT.

ID	Log_2_FC	Notes
**AN11049**	8.119	NmrA-like domain containing protein; predicted secondary metabolism gene cluster member
AN10650	6.821	Ortholog of *A*. *nidulans* FGSC A4: AN11203, and *A*. *fumigatus* Af293: Afu4g00520
**AN10044**	6.635	*mdpK*
**AN3568**	6.536	Protein of unknown function
**AN5430**	6.505	Protein of unknown function
**AN0146**	6.435	mdpC
AN3893	6.219	Protein of unknown function
**AN8105**	6.165	Putative NRPS-like enzyme; predicted secondary metabolism gene cluster member
**AN6314**	5.857	Has domain(s) with predicted nucleotide binding, oxidoreductase activity and role in metabolic process
AN7116	5.835	Protein of unknown function
AN6784	5.553	*xptA*
AN8101	5.519	Ortholog of *A*. *fumigatus* Af293: Afu5g02655, and *A*. *oryzae* RIB40: AO090102000386
AN3983	5.448	Ortholog of *A*. *wentii*: Aspwe1_0168245, and *A*. *clavatus* NRRL 1: ACLA_062140
AN11203	5.438	Ortholog of *A*. *nidulans* FGSC A4: AN10650, and *A*. *fumigatus* Af293: Afu4g00520
AN9290	5.428	Has domain(s) with predicted integral to membrane localization
**AN0149**	5.294	*mdpF*
**AN10023**	5.291	*mdpL*
**AN11037**	5.245	Predicted transmembrane transporter; predicted secondary metabolism gene cluster member
**AN1242**	5.225	Putative NRPS involved in nidulanin A biosynthesis; predicted backbone enzyme of the secondary metabolite gene cluster
AN8775	5.001	Ortholog of *Neosartorya fischeri* NRRL 181: NFIA_004310

*Genes that are up-regulated in both Δ*osaA veA1* and *veA* ^+^ WT are in bold.

**Table 4:** Top 20 down-regulated genes in Δ*osaA veA1* relative to *veA1* WT.

ID	Log_2_FC	Notes
AN7836	-9.872	Ortholog of *A*. *fumigatus* Af293: Afu7g01060, and *A*. *oryzae* RIB40: AO090003000833
AN7839	-9.739	Has domain(s) with predicted ATP binding, ATPase activity, coupled to transmembrane movement of substances activity, role in transmembrane transport and integral to membrane localization
AN11574	-9.614	Protein of unknown function
AN12330	-9.021	Protein of unknown function
AN7834	-8.786	Protein of unknown function
AN12331	-7.868	Putative PKS-like enzyme
**AN6476**	-7.423	Protein of unknown function
**AN6464**	-7.305	Has domain(s) with predicted hydrolase activity, acting on ester bonds activity and role in lipid metabolic process
AN5032	-6.489	Has domain(s) with predicted role in transmembrane transport and integral to membrane localization
**AN8609**	-6.396	Predicted glycosylphosphatidylinositol-anchored protein
AN11027	-6.061	Has domain(s) with predicted acyl-CoA hydrolase activity and role in acyl-CoA metabolic process
AN5558	-6.035	*prtA*
AN8969	-5.893	Has domain(s) with predicted cation binding, lysozyme activity and role in carbohydrate metabolic process, cell wall macromolecule catabolic process, peptidoglycan catabolic process
**AN7402**	-5.613	*glaB*
AN3996	-5.602	Has domain(s) with predicted methyltransferase activity and role in metabolic process
**AN2380**	-5.569	Protein of unknown function
AN5267	-5.402	*faeC*
AN7396	-5.382	*bglM*
AN7962	-5.247	*pepJ*
AN12183	-5.091	Protein of unknown function

*Genes that are down-regulated in both Δ*osaA veA1* and *veA* ^+^ WT are in bold.

**Table 5:** Top 20 down-regulated genes in *veA* ^+^ WT relative to *veA1* WT.

ID	Log_2_FC	Notes
**AN2380**	-6.560	Protein of unknown function
AN6798	-6.255	Has domain(s) with predicted catalytic activity and role in metabolic process
AN1567	-5.711	Ortholog of *A*. *fumigatus* Af293: Afu5g00910, and *Neosartorya fischeri* NRRL 181
**AN6476**	-4.876	Protein of unknown function
AN8476	-4.583	Has domain(s) with predicted aminopeptidase activity, dipeptidyl-peptidase activity and role in proteolysis
AN0011	-4.445	Possible pseudogene
AN2538	-4.311	Ortholog of *A*. *versicolor*: Aspve1_0026536 and *A*. *sydowii*: Aspsy1_0028980
**AN6464**	-4.245	Has domain(s) with predicted hydrolase activity, acting on ester bonds activity and role in lipid metabolic process
**AN7402**	-4.058	*glaB*
**AN8609**	-4.050	Predicted glycosylphosphatidylinositol-anchored protein
AN4817	-3.997	Has domain(s) with predicted role in transmembrane transport and integral to membrane localization
AN5315	-3.976	Ortholog of *A*. *nidulans* FGSC A4: AN7859, and *A*. *niger* CBS 513.88
AN2465	-3.785	Has domain(s) with predicted substrate-specific transmembrane transporter activity, role in transmembrane transport and integral to membrane localization
AN7870	-3.767	Ortholog of *A*. *fumigatus* Af293: Afu3g01650, Afu6g11970, and *Neosartorya fischeri* NRRL 181: NFIA_002730
AN6820	-3.726	hk-8-3
AN3205	-3.605	Putative aldehyde dehydrogenase; ortholog of *A*. *fumigatus* Afu4g02830
AN6470	-3.604	Protein with lysozyme activity, involved in carbohydrate catabolism
AN3218	-3.597	Ortholog of *A*. *oryzae* RIB40: AO090701000492 and *A*. *flavus* NRRL 3357: AFL2T_06113
AN4816	-3.554	Ortholog of *A*. *oryzae* RIB40: AO090020000221, and *A*. *versicolor*: Aspve1_0128410
AN2395	-3.505	Putative beta-glucuronidase with a predicted role in polysaccharide degradation

*Genes that are down-regulated in both Δ*osaA veA1* and *veA* ^+^ WT are in bold.

The majority of the top differentially expressed genes obtained for both VeA and OsaA are related to metabolism (Tables 2, 3, 4 and 5); including polyketide synthases (PKS), ATPases, non-ribosomal peptide synthases (NRPS), and a plethora of various enzymes. In *Aspergillus* several metabolites are involved in regulating sporulation levels [1, 8, 50, 51]. For instance, the *veA* **—|** *osaA* pathway is necessary for the proper expression of the *ivo* and *wA* clusters that play different roles in *A*. *nidulans* sporulation [52, 53]. Interestingly, OsaA down-regulates expression of the monodictyphenone gene cluster [54] through the *veA* **—|** *osaA* pathway. Genes within the monodictyphenone cluster were in the top 20 up-regulated genes in Δ*osaA veA1* (Table 2). Taken together, biosynthesis of several metabolites are subject to the OsaA-mediated orchestration.

### Expression analyses of developmental genes

**Fig 5:**
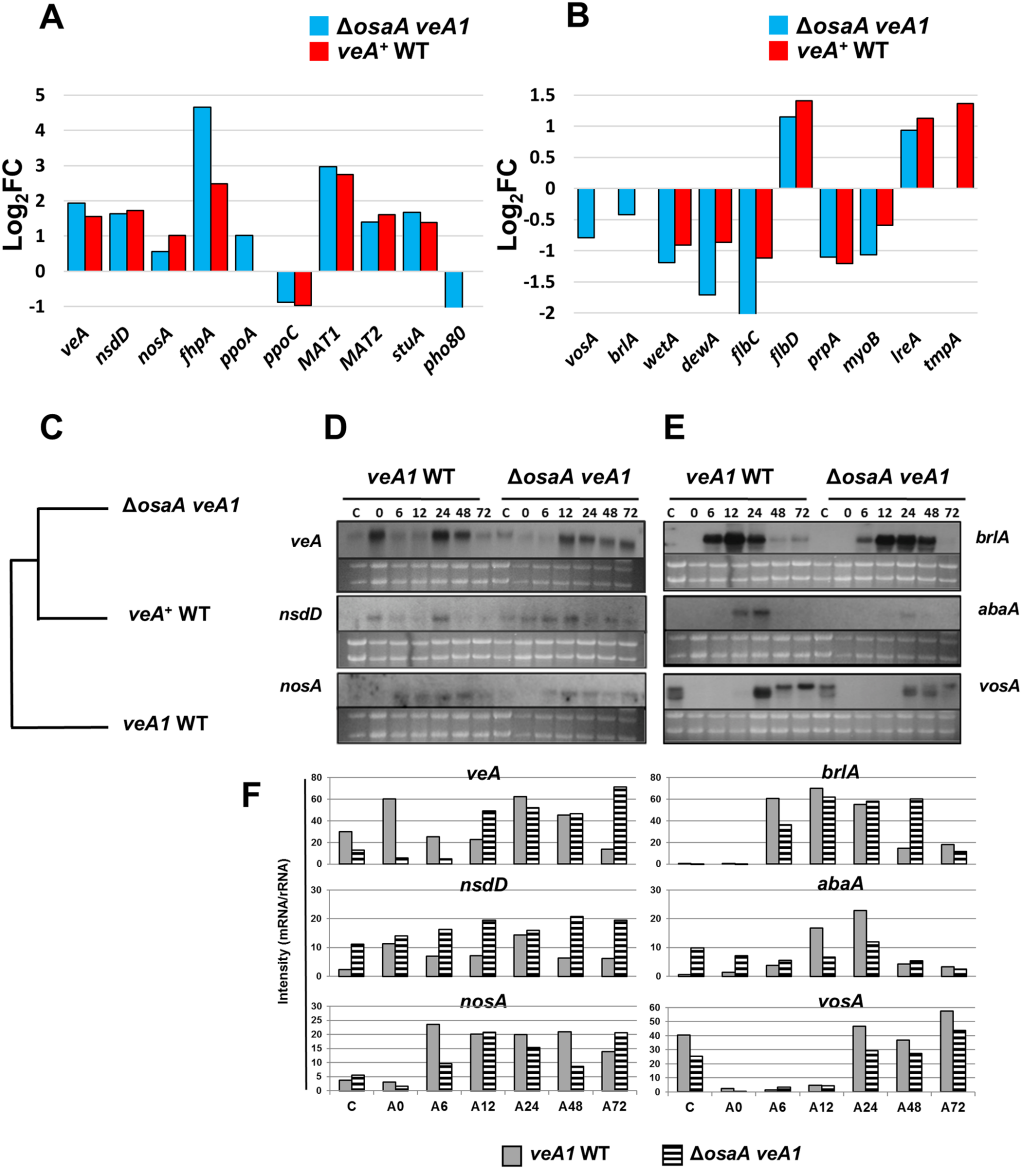
Influence of OsaA on expression of developmental genes. (A) A histogram showing log_2_FC values of select sexual regulators in *veA* ^*+*^ WT and Δ*osaA veA1* relative to *veA1* WT. (B) A histogram showing log_2_FC values of select asexual regulators in *veA* ^*+*^ WT and Δ*osaA veA1* relative to *veA1* WT. (C) Phylogenetic analysis of select developmental gene expression changes among *veA1* WT, *veA* ^*+*^ WT and *ΔosaA veA1* strains. Only those genes with significant changes in pair-wise comparisons (p-value < = 5%) were analyzed using their RPKM values for hierarchical clustering. (D) Northern blot showing mRNA accumulation levels of three sexual activators, *veA*, *nsdD*, and *nosA*, in *veA1* WT and Δ*osaA veA1*. (E) Northern blot showing mRNA accumulation levels of three asexual regulators, *brlA*, *abaA* and *vosA*, in *veA1* WT and Δ*osaA veA1*. (F) Densitometry analysis of the northern blot data shown in (Fig 5D and 5E). Band intensity was estimated in reference to the rRNA levels using imageJ software [46].

Relative to *veA1* WT, in both *veA* ^*+*^ WT and Δ*osaA veA1* strains the majority of sexual activators were up-regulated (Fig 5A and S1 Table) [8]. In contrast, many downstream asexual regulators (Fig 5B and S1 Table) were down-regulated in both *veA* ^*+*^ WT and Δ*osaA veA1* strains compared to *veA1* WT [3, 55, 56]. These findings correspond to the distinct developmental phenotypes of *veA* ^*+*^ WT and Δ*osaA veA1*, which show significantly higher levels of cleistothecia and much reduced levels of conidiation in comparison to *veA1* WT. The hierarchical clustering-based of all developmental gene expression available in AspGD [34, 35] shows that Δ*osaA veA1* and *veA* ^*+*^ WT cluster in one group separated from the *veA1* WT clade. Hence, loss of *osaA* has competency in suppressing the *veA1* mutation’s effect on developmental gene expression (Fig 5C). S1 Table shows the list of differentially expressed development-related genes.

To confirm the RNA-Seq data and to further examine the expression pattern of developmental genes, we examined mRNA accumulation levels, via Northern blot, in Δ*osaA veA1* comparing to *veA1* WT (Fig 5D and 5E). Similar to the RNA-Seq data, mRNA levels of upstream sexual activators (*veA* [14], *nsdD* [57]) were overall higher in Δ*osaA veA1* in comparison to *veA1* WT (Fig 5D). The *veA* transcript in Δ*osaA veA1* accumulated 12 h earlier and it was detectable up to 72 h, whereas in *veA1* WT the transcript signal disappeared at 72 h. However, the downstream positive sexual regulator *nosA* [58] exhibited varying mRNA levels through out the tested time points (Fig 5D). One the other hand, the asexual regulators (*brlA* [5], *abaA* [59] and *vosA* [11]) showed an overall reduced and altered levels of mRNA accumulation in Δ*osaA veA1* comparing to *veA1* WT (Fig 5E). The *brlA* transcript in Δ*osaA veA1* showed lower accumulation levels at 6 h and its signal stays high at 48 h (Fig 5E), whereas in *veA1* WT the *brlA* transcript started accumulating from 6 h and disappeared at 48 h.

VeA and NsdD are proposed to down-regulate *brlA* [13, 60]. Moreover, the early accumulation of the *veA* transcript (Fig 5E) is suggested to inhibit the conidiophore formation through interfering with the competence time required to initiate conidiation [45, 61]. Accordingly, the lower *brlA* mRNA accumulation levels and the reduced conidiation levels in the Δ*osaA* mutants might be caused by the higher and early accumulation levels of *nsdD* and *veA* transcripts, respectively.

### WOPR might be functionally conserved in Aspergillus

**Fig 6:**
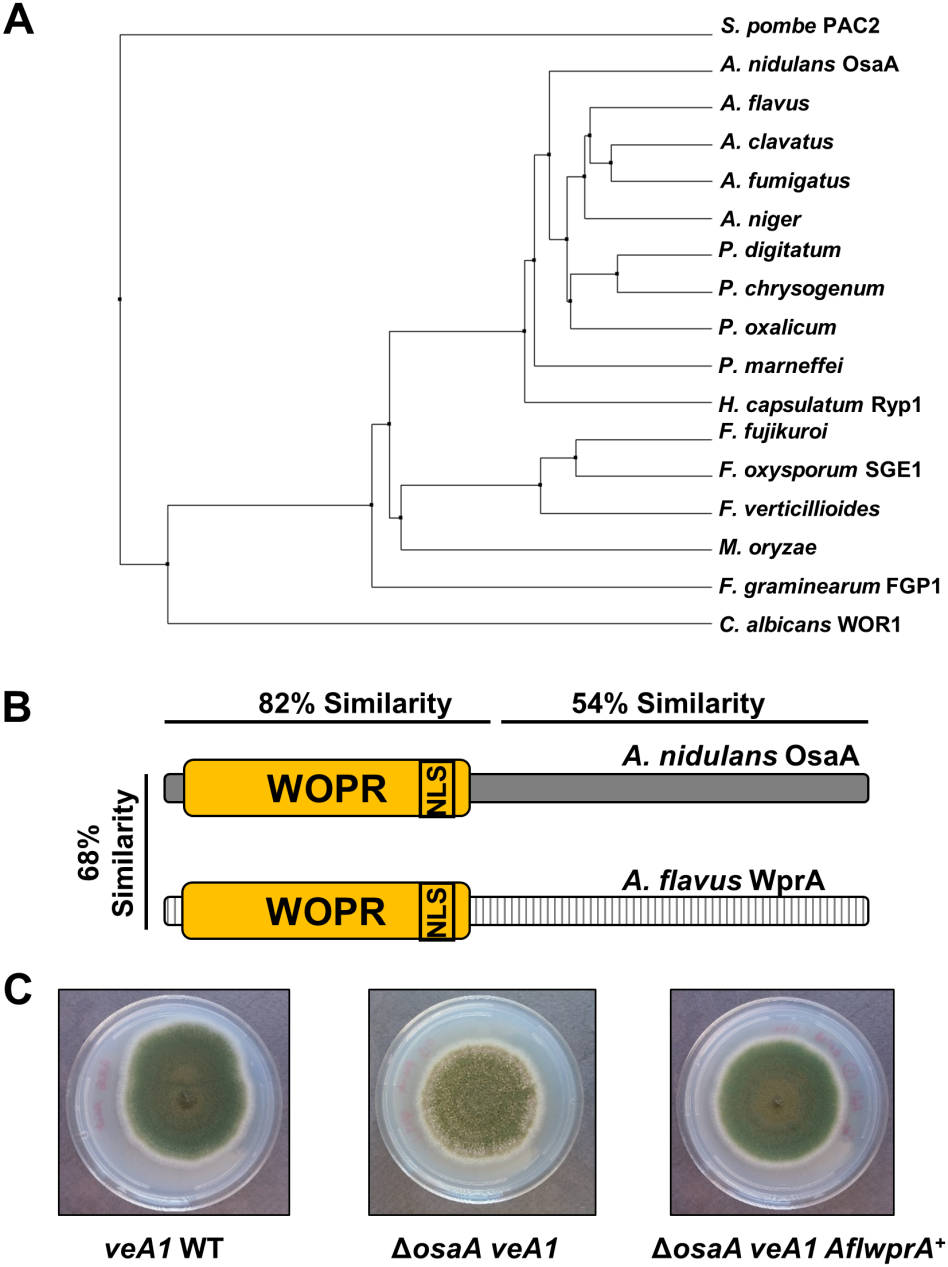
*A*. *flavus wprA* can complement *ΔosaA* in *A*. *nidulans*. (A) A phylogenetic tree of the WOPR proteins homologous to OsaA in several fungi. *A*. *nidulans* OsaA (AN6578), *A*. *flavus* (AFL2T_08419), *A*. *fumigatus* (Afu5g12960), *A*. *clavatus* (XP_001270095.1), *A*. *niger* (XP_001396650.1), *H*. *capsulatum* Ryp1 (ABX74945.1), *F*. *oxysporum* Sge1 (AGA55574.1), *F*. *graminearum* Fgp1 (I1S5P3), *P*. *digitatum* (EKV12249.1), *P*. *chrysogenum* (XP_002563706.1), *P*. *oxalicum* (EPS28657.1), *P*. *marneffei* (XP_002153295.1), *F*. *fujikuroi* (S0E3H0), *F*. *verticillioides* (W7MPI5.1), *M*. *oryzae* (XP_003713871.1), *C*. *albicans* Wor1 (Q5AP80) and *S*. *pombe* Pac2 (BAC54908.1). (B) AflWprA and OsaA polypeptides showing the amino acid similarity. Yellow box indicates the WOPR domain, NLS: nuclear localization sequence. (C) Colony photographs showing *veA1* WT, *osaA* mutant (Δ*osaA veA1*) and cross-complementation (Δ*osaA veA1 AflwprA*) strains.

Pair-wise alignment of the *Aspergillus* WOPR proteins (using only the ones that showed the highest similarity score to *A*. *nidulans* OsaA) revealed a high homology amongst all *Aspergillus* WOPR proteins even at the less conserved C-terminus (data not shown). *Aspergillus* WOPRs showed higher than 80% aa similarity, except, *A*. *nidulans* OsaA that showed the lowest similarity index comparing to all the examined *Aspergillus* WOPRs (~68%). The C-terminal region showed above 50% homology among all aspergilli WOPRs. We constructed a phylogenetic tree using only the WOPR domain in various fungal species (Fig 6A). *Aspergillus* WOPRs cluster with the *C*. *albicans* Wor1 clade indicating that *Aspergillus* OsaA homologs are more related to Wor1 than to the closely related *S*. *pombe* Pac2 protein [20, 62].

Through BLAST search (http://www.ncbi.nlm.nih.gov) we were able to identify OsaA’s homolog in *A*. *flavus* (AflWprA). OsaA and AflWprA proteins share 68% aa similarity. The homology percentage of the N-terminal region containing the WOPR domain is 82%, whereas the C-terminal region shows 54% similarity (Fig 6B). To test whether OsaA and AflWprA are functionally conserved, we carried out a cross-complementation experiment by introducing an *AflwprA* genomic DNA fragment to a Δ*osaA* strain, yielding the Δ*osaA veA1 AflwprA* ^*+*^ strains. As shown in Fig 6C, an example of such strains exhibited restored balance of sexual and asexual development to *veA1* WT. These results indicate that WOPRs in *Aspergillus* are likely functionally conserved. The downstream gene targets for each WOPR protein, however, might be different in each species.

## Discussion

The WOPR-domain family of global regulators (WOPRs) play critical roles in almost all fungal species. Despite the critical biological roles of WOPRs, no clear bioinformatics predictions of known-functional domains could have been made. For the past decade, extensive studies have been carried out to reveal the molecular mechanisms of WOPRs function. The WOPR domain consists of two regions (80 and 50 aa) conserved across many fungal species, separated by a non-conserved linker region with variable length (25 ~ 100 aa) [17, 18, 44]. Importantly, recent studies have solved the crystal structures of two WOPRs, *S*. *cerevisiae* WOPR YHR177w [44] and the *C*. *albicans* Wor1 segments [20], in complex with dsDNA containing a consensus binding sequence. Both studies show that the two conserved regions are tightly bound to each other through a β-sheet, with β-strands from one conserved region interdigitated with those from the other, with the linker looped out away from the DNA [17]. However, Lohse et al found that the most important contacts with DNA are made by an adjacent loop (contributed by Arg1), which is inserted into an especially narrow minor groove where it makes base-specific and backbone contacts. These studies unveil that the WOPR domain represents a new family of fungi-specific DNA-binding proteins, one with key roles for fungal morphogenesis and pathogenesis.

The protein-DNA interactions are essential for *WOR1* transcriptional regulation and white-to-opaque switching. The best studied Wor1 (white-opaque switching regulator 1) protein, a master regulator of the white-opaque switching in *Candida albicans*, binds to a consensus 9-nucleotide core motif (TTAAAGTTT) in three different fungal species, *C*. *albicans* Wor1, *H*. *capsulatum* Ryp1, and *Saccharomyces ceriviseae* Mit1, indicating conservation of the WOPR domain–DNA sequence interactions over a period of 600 million to 1.2 billion years of divergence [18, 24, 44]. These WOPRs exhibit transcriptional regulation of downstream gene targets by directly interacting with the *cis*-regulatory elements.

In this study, we show that the *A*. *nidulans* WOPR regulator OsaA is an orchestrator of development. OsaA functions as an upstream repressor of sexual development. Sexual fruiting is subject to a tight regulation due to its high metabolic and structural costs [1, 2, 4, 8]. In addition to OsaA, a number of sexual fruiting repressors have previously been characterized in *A*. *nidulans*. RosA is an upstream transcriptional factor that represses sexual development under low glucose conditions [63]. Light inhibits sexual development mainly through the activity of the red light-sensing phytochrome FphA [64]. G-protein signaling components, RasA (GTPase) [65] and GprD (G protein coupled receptor) [66], also play critical roles in repressing sexual development. PpoC, an oxylipin biosynthetic oxygenase, represses sexual development through the biosynthesis of the 10’ oxylipins that signal the deactivation of sexual sporulation [67]. Taken together, regulating sexual fruiting requires a dynamic network that is able to integrate intrinsic signals with surrounding extrinsic cues.

**Fig 7:**
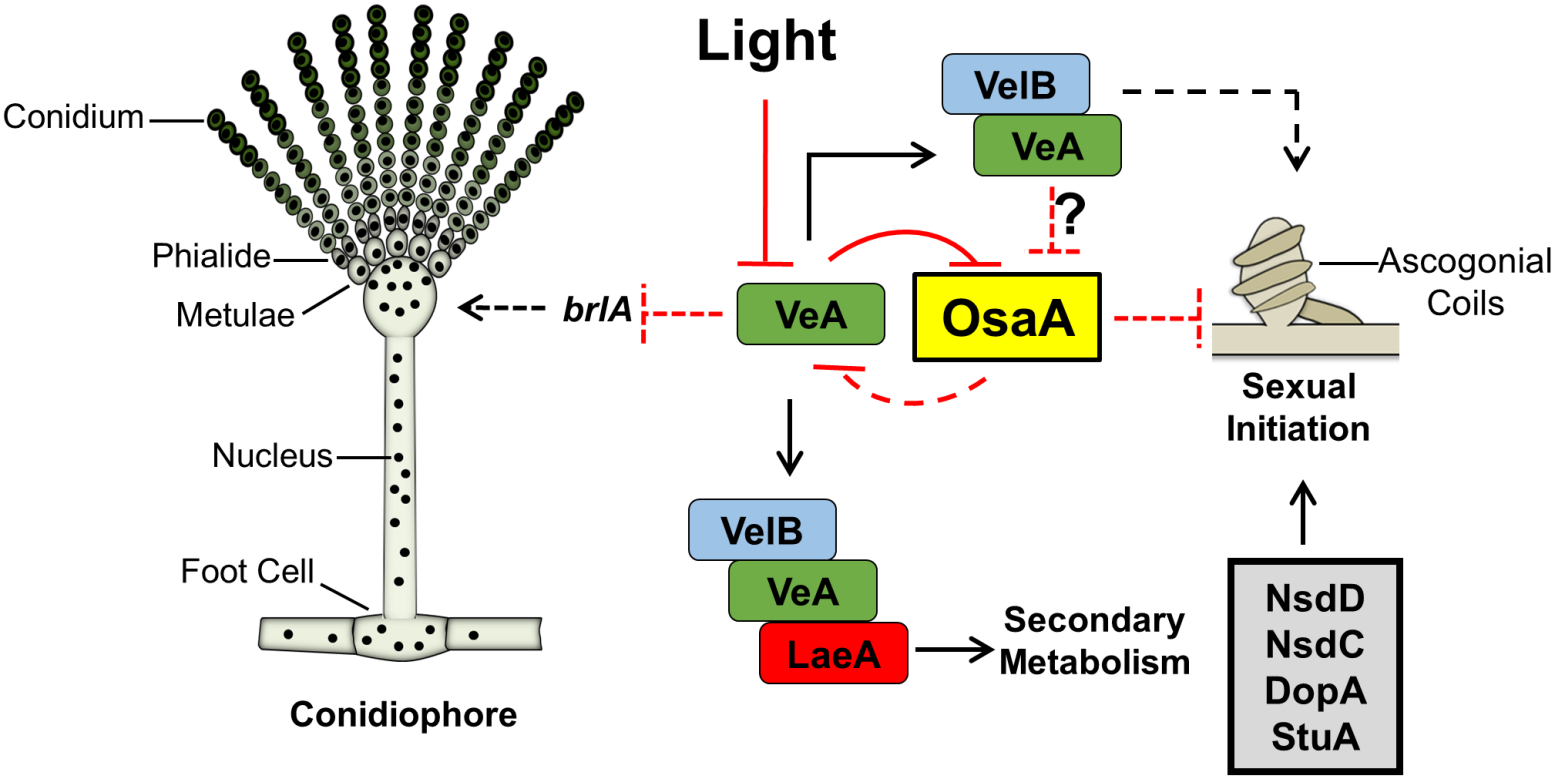
Genetic model for balanced development in *A*. *nidulans*. Blunt ended lines indicate negative regulation, arrowheads indicate positive regulation, and dashed lines indicate potential/indirect relationship. See the main text.

Through genetic and transcriptomic approaches we found that *osaA* functions downstream of the *velvet* regulator *veA*; i.e., the loss of *osaA* is sufficient to suppress the *veA1* mutation. It is proposed that VeA is required to remove OsaA’s repressive effects on sexual development. Both *velvets* and WOPRs are fungi-specific regulators that are found in a large number of fungal genomes [9, 10, 17, 18, 68]. The *veA1* mutation interferes with VeA nuclear translocation due to the lack of NLS [16]. The *osaA* gene exhibited higher mRNA accumulation levels in *veA1* WT, suggesting a possibility of VeA’s role in down-regulating transcription of *osaA*. As a consequence, sexual development is significantly reduced in *veA1* WT (Fig 2). The VeA regulatory pathways for development and SM are separated. The VelB-VeA dimer is involved in regulating sexual development, while the VelB-VeA-LaeA complex is required for SM [10, 47]. The loss of *osaA* did not affect the levels of the mycotoxin sterigmatocystin production in comparison to WT. Thus, OsaA is possibly regulated through the VelB-VeA dimer, or by an alternative VeA-regulated genetic pathway (Fig 7).

Our data show that virtually all critical asexual activators were down-regulated, while most known activators of sexual development were relatively up-regulated in both Δ*osaA veA1* and *veA* ^*+*^ WT (Fig 5A)[8]. This shift in gene expression relative to *veA1* WT might explain the basis for the higher levels of sexual sporulation in *veA* ^*+*^ WT and Δ*osaA veA1* strains. Up-regulation of many sexual activators, which in turn have a global suppressive effect on conidiation, explains the observed Δ*osaA* phenotype [1, 3]. OsaA also appears to down-regulate *veA* mRNA levels; the *veA* transcript exhibits earlier and higher accumulation levels in Δ*osaA veA1* comparing to WT. Early accumulation of *veA* transcript might interfere with the competence time required to initiate asexual development [45, 61]. This could explain: 1) the suppressed conidiation levels in Δ*osaA* strains comparing to WT, and 2) reduced *brlA* mRNA levels at early asexual development in Δ*osaA veA1* comparing to WT. These findings propose that *osaA* and *veA* may have a cross-repression genetic relationship that controls development and maintains a tight balance of asexual-to-sexual spore ratio (Fig 7).

The sporulation block represented by the *fluffy* phenotype obtained from the multi-copy expression of *osaA* is likely triggered by mis-regulation of the finely-tuned spatial and temporal genetic control of downstream morphologic and metabolic pathways. This speculation is based on the genome-wide expression changes of more than 2,500 genes by the deletion of *osaA*. Furthermore, our data show that *osaA* is not involved in the FluG—|SfgA main conidiation activating pathway. Taken together, instead of directly affecting asexual development, OsaA influences conidiation by repressing sexual development. On the other hand, the transcriptomic analysis shows that the *veA* **—|** *osaA* pathway might not be specific to morphological development, and might also regulate genes related to metabolism. This promiscuous functional behavior is due to the fact that development is a type of fungal morphogenesis, which requires both a significant epigenetic and biochemical shifts. Therefore, the genes related to processes other than development are possibly necessary to cater to the need of differentiated reproductive structures.

Each WOPR protein plays a distinct role in the fungal species it belongs to [18–20, 22, 23, 69]. We show that *AflwprA* was sufficient to repress enhanced sexual development in Δ*osaA veA1* strain and restore the sporulation ratios back to that of *veA1* WT (Fig 6C). This cross-complementation experiment suggests that WOPRs in *Aspergillus* are functionally conserved and might recognize the same *cis*-regulatory elements, despite the 450 million years’ of divergence between *A*. *nidulans* and *A*. *flavus* [70]. This further proposes an idea that the differential roles of WOPR controlling species-specific biology might be due to the modifications in each fungal genome rather than changes in the WOPR domain.

In summary, through genetic and transcriptomic studies we found that OsaA is a key upstream regulator of development primarily acting as a repressor of sexual fruiting in *A*. *nidulans*. OsaA acts downstream of the *velvet* regulator *veA* and orchestrates the developmental balance and progression. A major part of VeA role in activating sexual fruiting is likely removing OsaA’s repressive effects. In *Aspergillus*, the function of each WOPR protein is species dependent, and variations in WOPR roles are due to variations in the fungal genomes rather than variations in the conserved WOPR domain. Further studies are in progress to understand OsaA’s role in regulating development and SM in other *Aspergillus* species, and to determine direct targets of OsaA.

## Supporting Information

S1 Fig High quality RNA-Seq data sets. Scattered plot showing the correlation levels among triplicates of each sample. The correlation coefficient *R* for Δ*osaA veA1* was > 0.97, *veA1* WT was > 0.97, and *veA* ^*+*^ WT was > 0.97, all with p-value less than 0.01, indicating the high quality of the RNA-Seq data sets.
(TIF)

S2 Fig Representation of the numbers of genes by sequenced RNA. FPKM obtained for Δ*osaA veA1*, *veA1* WT, and *veA* ^*+*^ WT strains are mapped to 10,536, 10,428 and 10,514 genes, respectively, representing 96.3%, 95.3% and 96% coverage of a total of 10,943 genes predicted by AspGD.
(TIF)

S3 Fig Validation of the *osaA* deletion by RNA-Seq. A snapshot from Integrative Genomics Viewer (IGV) software showing the *osaA* locus (AN6578) in Δ*osaA veA1*, *veA1* WT, and *veA* ^*+*^ WT strains.
(TIF)

S4 Fig A diagram showing the three overlapping *osaA* transcripts. Open box represents *osaA* locus; grey box represents ORF; arrow line represents transcript; open triangle in transcript ~1kb indicates alternative splicing.
(TIF)

S5 Fig Multidimensional scaling plot showing correlations among strains. The overall transcriptomic profiles of Δ*osaA veA1*, *veA1* WT, and *veA* ^*+*^ WT strains examined by a two-dimensional plot. Black circles indicate replicates.
(TIF)

S1 Table A list of differentially expressed development-related genes in both *veA* ^*+*^ WT and Δ*osaA veA1* relative to *veA1* WT. (DOCX)

S2 Table Oligonucleotides used in this study. (DOCX)

## References

[1] Ugalde U, Rodriguez-Urra AB. The Mycelium Blueprint: insights into the cues that shape the filamentous fungal colony. Applied microbiology and biotechnology. 2014;98(21):8809–19. 10.1007/s00253-014-6019-6 . 25172134

[2] Yu JH. Regulation of Development in *Aspergillus nidulans* and *Aspergillus fumigatus* . Mycobiology. 2010;38(4):229–37. Epub 2010/12/01. 10.4489/MYCO.2010.38.4.229 23956662 PMC3741515

[3] Park HS, Yu JH. Genetic control of asexual sporulation in filamentous fungi. Curr Opin Microbiol. 2012;15(6):669–77. Epub 2012/10/25. 10.1016/j.mib.2012.09.006 . 23092920

[4] Adams TH, Wieser JK, Yu JH. Asexual sporulation in *Aspergillus nidulans* . Microbiol Mol Biol Rev. 1998;62(1):35–54. Epub 1998/04/08. 9529886 10.1128/mmbr.62.1.35-54.1998 PMC98905

[5] Adams TH, Boylan MT, Timberlake WE. *brlA* is necessary and sufficient to direct conidiophore development in *Aspergillus nidulans* . Cell. 1988;54(3):353–62. Epub 1988/07/29. . 3293800 10.1016/0092-8674(88)90198-5

[6] Chang YC, Timberlake WE. Identification of Aspergillus *brlA* response elements (BREs) by genetic selection in yeast. Genetics. 1993;133(1):29–38. Epub 1993/01/01. 8417986 10.1093/genetics/133.1.29 PMC1205295

[7] Sohn KT, Yoon KS. Ultrastructure Study of the Cleistothecium Dvevelopment in *Aspergillus nidulans* . microbiology. 2002;30(3):117–27.

[8] Dyer PS, O'Gorman CM. Sexual development and cryptic sexuality in fungi: insights from *Aspergillus* species. FEMS Microbiol Rev. 2012;36(1):165–92. Epub 2011/11/19. 10.1111/j.1574-6976.2011.00308.x . 22091779

[9] Bayram O, Braus GH. Coordination of secondary metabolism and development in fungi: the velvet family of regulatory proteins. FEMS Microbiol Rev. 2012;36(1):1–24. Epub 2011/06/11. 10.1111/j.1574-6976.2011.00285.x . 21658084

[10] Bayram O, Krappmann S, Ni M, Bok JW, Helmstaedt K, Valerius O, et al VelB/VeA/LaeA complex coordinates light signal with fungal development and secondary metabolism. Science. 2008;320(5882):1504–6. Epub 2008/06/17. 10.1126/science.1155888 . 18556559

[11] Ni M, Yu JH. A novel regulator couples sporogenesis and trehalose biogenesis in *Aspergillus nidulans* . PLoS One. 2007;2(10):e970 Epub 2007/10/04. 10.1371/journal.pone.0000970 17912349 PMC1978537

[12] Park HS, Nam TY, Han KH, Kim SC, Yu JH. VelC positively controls sexual development in *Aspergillus nidulans* . PLoS One. 2014;9(2):e89883 10.1371/journal.pone.0089883 24587098 PMC3938535

[13] Kato N, Brooks W, Calvo AM. The expression of sterigmatocystin and penicillin genes in *Aspergillus nidulans* is controlled by *veA*, a gene required for sexual development. Eukaryotic cell. 2003;2(6):1178–86. 14665453 10.1128/EC.2.6.1178-1186.2003 PMC326641

[14] Kim H, Han K, Kim K, Han D, Jahng K, Chae K. The *veA* gene activates sexual development in *Aspergillus nidulans* . Fungal Genet Biol. 2002;37(1):72–80. Epub 2002/09/12. . 12223191 10.1016/s1087-1845(02)00029-4

[15] Yager LN. Early developmental events during asexual and sexual sporulation in *Aspergillus nidulans* . Biotechnology. 1992;23:19–41. . 1504597

[16] Kafer E. Origins of translocations in *Aspergillus nidulans* . Genetics. 1965;52(1):217–32. 5857597 10.1093/genetics/52.1.217 PMC1210839

[17] Lohse MB, Rosenberg OS, Cox JS, Stroud RM, Finer-Moore JS, Johnson AD. Structure of a new DNA-binding domain which regulates pathogenesis in a wide variety of fungi. Proc Natl Acad Sci U S A. 2014;111(29):10404–10. Epub 2014/07/06. 10.1073/pnas.1410110111 24994900 PMC4115540

[18] Lohse MB, Zordan RE, Cain CW, Johnson AD. Distinct class of DNA-binding domains is exemplified by a master regulator of phenotypic switching in Candida albicans. Proc Natl Acad Sci U S A. 2010;107(32):14105–10. Epub 2010/07/28. 10.1073/pnas.1005911107 20660774 PMC2922561

[19] Nguyen VQ, Sil A. Temperature-induced switch to the pathogenic yeast form of Histoplasma capsulatum requires Ryp1, a conserved transcriptional regulator. Proc Natl Acad Sci U S A. 2008;105(12):4880–5. Epub 2008/03/15. 10.1073/pnas.0710448105 18339808 PMC2290814

[20] Huang G, Wang H, Chou S, Nie X, Chen J, Liu H. Bistable expression of WOR1, a master regulator of white-opaque switching in Candida albicans. Proc Natl Acad Sci U S A. 2006;103(34):12813–8. Epub 2006/08/15. 16905649 10.1073/pnas.0605270103 PMC1540355

[21] Liu OW, Chun CD, Chow ED, Chen C, Madhani HD, Noble SM. Systematic genetic analysis of virulence in the human fungal pathogen *Cryptococcus neoformans* . Cell. 2008;135(1):174–88. 10.1016/j.cell.2008.07.046 18854164 PMC2628477

[22] Jonkers W, Dong Y, Broz K, Kistler HC. The Wor1-like protein Fgp1 regulates pathogenicity, toxin synthesis and reproduction in the phytopathogenic fungus Fusarium graminearum. PLoS Pathog. 2012;8(5):e1002724 Epub 2012/06/14. 10.1371/journal.ppat.1002724 22693448 PMC3364952

[23] Michielse CB, van Wijk R, Reijnen L, Manders EM, Boas S, Olivain C, et al The nuclear protein Sge1 of *Fusarium oxysporum* is required for parasitic growth. PLoS Pathog. 2009;5(10):e1000637 Epub 2009/10/24. 10.1371/journal.ppat.1000637 19851506 PMC2762075

[24] Beyhan S, Gutierrez M, Voorhies M, Sil A. A temperature-responsive network links cell shape and virulence traits in a primary fungal pathogen. PLoS Biol. 2013;11(7):e1001614 Epub 2013/08/13. 10.1371/journal.pbio.1001614 23935449 PMC3720256

[25] Pontecorvo G, Roper JA, Hemmons LM, Macdonald KD, Bufton AW. The genetics of *Aspergillus nidulans* . Adv Genet. 1953;5:141–238. . 13040135 10.1016/s0065-2660(08)60408-3

[26] Kafer E. Meiotic and mitotic recombination in *Aspergillus* and its chromosomal aberrations. Adv Genet. 1977;19:33–131. . 327767 10.1016/s0065-2660(08)60245-x

[27] Seo JA, Guan Y, Yu JH. Suppressor mutations bypass the requirement of fluG for asexual sporulation and sterigmatocystin production in *Aspergillus nidulans* . Genetics. 2003;165(3):1083–93. 14668366 10.1093/genetics/165.3.1083 PMC1462808

[28] Ni M, Rierson S, Seo JA, Yu JH. The pkaB gene encoding the secondary protein kinase A catalytic subunit has a synthetic lethal interaction with pkaA and plays overlapping and opposite roles in *Aspergillus nidulans* . Eukaryotic cell. 2005;4(8):1465–76. 16087751 10.1128/EC.4.8.1465-1476.2005 PMC1214532

[29] Brown SH, Zarnowski R, Sharpee W, Keller N. Morphological transitions governed by density dependence and lipoxygenase activity in *Aspergillus flavus* . Applied and environmental microbiology. 2008;74(18):5674–85. 10.1128/AEM.00565-08 18658287 PMC2547031

[30] Osherov N, Mathew J, May GS. Polarity-defective mutants of *Aspergillus nidulans* . Fungal Genet Biol. 2000;31(3):181–8. Epub 2001/03/29. 10.1006/fgbi.2000.1236 . 11273680

[31] Broad Institute of Harvard and MIT (http://www.broadinstitute.org/).

[32] Yu JH, Hamari Z, Han KH, Seo JA, Reyes-Dominguez Y, Scazzocchio C. Double-joint PCR: a PCR-based molecular tool for gene manipulations in filamentous fungi. Fungal Genet Biol. 2004;41(11):973–81. Epub 2004/10/07. 10.1016/j.fgb.2004.08.001 . 15465386

[33] Han KH, Seo JA, Yu JH. Regulators of G-protein signalling in *Aspergillus nidulans*: RgsA downregulates stress response and stimulates asexual sporulation through attenuation of GanB (Galpha) signalling. Molecular microbiology. 2004;53(2):529–40. . 15228532 10.1111/j.1365-2958.2004.04163.x

[34] Arnaud MB, Cerqueira GC, Inglis DO, Skrzypek MS, Binkley J, Chibucos MC, et al The *Aspergillus* Genome Database (AspGD): recent developments in comprehensive multispecies curation, comparative genomics and community resources. Nucleic Acids Res. 2012;40(Database issue):D653–9. 10.1093/nar/gkr875 22080559 PMC3245136

[35] Cerqueira GC, Arnaud MB, Inglis DO, Skrzypek MS, Binkley G, Simison M, et al The *Aspergillus* Genome Database: multispecies curation and incorporation of RNA-Seq data to improve structural gene annotations. Nucleic Acids Res. 2014;42(Database issue):D705–10. 10.1093/nar/gkt1029 24194595 PMC3965050

[36] Liao Y, Smyth GK, Shi W. The Subread aligner: fast, accurate and scalable read mapping by seed-and-vote. Nucleic Acids Res. 2013;41(10):e108 10.1093/nar/gkt214 23558742 PMC3664803

[37] Ritchie ME, Phipson B, Wu D, Hu Y, Law CW, Shi W, et al limma powers differential expression analyses for RNA-sequencing and microarray studies. Nucleic Acids Res. 2015;43(7):e47 10.1093/nar/gkv007 . 25605792 PMC4402510

[38] Trapnell C, Williams BA, Pertea G, Mortazavi A, Kwan G, van Baren MJ, et al Transcript assembly and quantification by RNA-Seq reveals unannotated transcripts and isoform switching during cell differentiation. Nat Biotechnol. 2010;28(5):511–5. 10.1038/nbt.1621 20436464 PMC3146043

[39] Trapnell C, Hendrickson DG, Sauvageau M, Goff L, Rinn JL, Pachter L. Differential analysis of gene regulation at transcript resolution with RNA-seq. Nat Biotechnol. 2013;31(1):46–53. 10.1038/nbt.2450 23222703 PMC3869392

[40] Roberts A, Trapnell C, Donaghey J, Rinn JL, Pachter L. Improving RNA-Seq expression estimates by correcting for fragment bias. Genome Biol. 2011;12(3):R22 10.1186/gb-2011-12-3-r22 21410973 PMC3129672

[41] Roberts A, Pimentel H, Trapnell C, Pachter L. Identification of novel transcripts in annotated genomes using RNA-Seq. Bioinformatics. 2011;27(17):2325–9. 10.1093/bioinformatics/btr355 . 21697122

[42] Lee BN, Adams TH. The *Aspergillus nidulans fluG* gene is required for production of an extracellular developmental signal and is related to prokaryotic glutamine synthetase I. Genes Dev. 1994;8(6):641–51. . 7926755 10.1101/gad.8.6.641

[43] Seo JA, Guan Y, Yu JH. FluG-dependent asexual development in *Aspergillus nidulans* occurs via derepression. Genetics. 2006;172(3):1535–44. Epub 2006/01/03. 10.1534/genetics.105.052258 16387865 PMC1456305

[44] Cain CW, Lohse MB, Homann OR, Sil A, Johnson AD. A conserved transcriptional regulator governs fungal morphology in widely diverged species. Genetics. 2012;190(2):511–21. Epub 2011/11/19. 10.1534/genetics.111.134080 22095082 PMC3276625

[45] Champe SP, Kurtz MB, Yager LN, Butnick NJ, Axelrod DE. Spore formation in *Aspergillus nidulans*: competence and other developmental processes. The fungal spore: morphogenetic controls. 1981:255–76.

[46] Schneider CA, Rasband WS, Eliceiri KW. NIH Image to ImageJ: 25 years of image analysis. Nat Methods. 2012;9(7):671–5. . 22930834 10.1038/nmeth.2089 PMC5554542

[47] Park HS, Ni M, Jeong KC, Kim YH, Yu JH. The role, interaction and regulation of the velvet regulator VelB in *Aspergillus nidulans* . PLoS One. 2012;7(9):e45935 Epub 2012/10/11. 10.1371/journal.pone.0045935 23049895 PMC3457981

[48] Barron G. *Emericella nidulans*—cleistothecium with hulle cells. University of Guelph 2013.

[49] Geiser DM, Timberlake WE, Arnold ML. Loss of meiosis in *Aspergillus* . Mol Biol Evol. 1996;13(6):809–17. Epub 1996/07/01. . 8754217 10.1093/oxfordjournals.molbev.a025641

[50] Gerke J, Bayram O, Feussner K, Landesfeind M, Shelest E, Feussner I, et al Breaking the silence: protein stabilization uncovers silenced biosynthetic gene clusters in the fungus *Aspergillus nidulans* . Applied and environmental microbiology. 2012;78(23):8234–44. Epub 2012/09/25. 10.1128/AEM.01808-12 23001671 PMC3497355

[51] Rodriguez-Urra AB, Jimenez C, Nieto MI, Rodriguez J, Hayashi H, Ugalde U. Signaling the induction of sporulation involves the interaction of two secondary metabolites in *Aspergillus nidulans* . ACS chemical biology. 2012;7(3):599–606. Epub 2012/01/12. 10.1021/cb200455u . 22234162

[52] Clutterbuck AJ. The genetics of conidiophore pigmentation in *Aspergillus nidulans* . Journal of general microbiology. 1990;136(9):1731–8. . 2283502 10.1099/00221287-136-9-1731

[53] Mayorga ME, Timberlake WE. The developmentally regulated *Aspergillus nidulans wA* gene encodes a polypeptide homologous to polyketide and fatty acid synthases. Mol Gen Genet. 1992;235(2–3):205–12. . 1465094 10.1007/BF00279362

[54] Bok JW, Chiang YM, Szewczyk E, Reyes-Dominguez Y, Davidson AD, Sanchez JF, et al Chromatin-level regulation of biosynthetic gene clusters. Nat Chem Biol. 2009;5(7):462–4. 10.1038/nchembio.177 19448638 PMC2891026

[55] Kwon NJ, Garzia A, Espeso EA, Ugalde U, Yu JH. FlbC is a putative nuclear C2H2 transcription factor regulating development in *Aspergillus nidulans* . Molecular microbiology. 2010;77(5):1203–19. Epub 2010/07/14. 10.1111/j.1365-2958.2010.07282.x . 20624219

[56] Krijgsheld P, Bleichrodt R, Van Veluw G, Wang F, Müller W, Dijksterhuis J, et al Development in *Aspergillus* . Studies in mycology. 2013;74:1–29. 10.3114/sim0006 23450714 PMC3563288

[57] Han KH, Han KY, Yu JH, Chae KS, Jahng KY, Han DM. The *nsdD* gene encodes a putative GATA-type transcription factor necessary for sexual development of *Aspergillus nidulans* . Molecular microbiology. 2001;41(2):299–309. Epub 2001/08/08. . 11489119 10.1046/j.1365-2958.2001.02472.x

[58] Vienken K, Fischer R. The Zn(II)2Cys6 putative transcription factor NosA controls fruiting body formation in *Aspergillus nidulans* . Molecular microbiology. 2006;61(2):544–54. Epub 2006/06/20. . 16780567 10.1111/j.1365-2958.2006.05257.x

[59] Andrianopoulos A, Timberlake WE. The *Aspergillus nidulans abaA* gene encodes a transcriptional activator that acts as a genetic switch to control development. Mol Cell Biol. 1994;14(4):2503–15. Epub 1994/04/01. 8139553 10.1128/mcb.14.4.2503-2515.1994 PMC358618

[60] Lee MK, Kwon NJ, Choi JM, Lee IS, Jung S, Yu JH. NsdD is a key repressor of asexual development in *Aspergillus nidulans* . Genetics. 2014;197(1):159–73. 10.1534/genetics.114.161430 24532783 PMC4012476

[61] Axelrod DE. Kinetics of differentiation of conidiophores and conidia by colonies of *Aspergillus nidulans* . Journal of general microbiology. 1972;73(1):181–4. . 4569577 10.1099/00221287-73-1-181

[62] Kunitomo H, Sugimoto A, Wilkinson CR, Yamamoto M. Schizosaccharomyces pombe pac2+ controls the onset of sexual development via a pathway independent of the cAMP cascade. Curr Genet. 1995;28(1):32–8. Epub 1995/06/01. . 8536311 10.1007/BF00311879

[63] Vienken K, Scherer M, Fischer R. The Zn(II)2Cys6 putative *Aspergillus nidulans* transcription factor repressor of sexual development inhibits sexual development under low-carbon conditions and in submersed culture. Genetics. 2005;169(2):619–30. Epub 2004/11/03. 15520269 10.1534/genetics.104.030767 PMC1449130

[64] Blumenstein A, Vienken K, Tasler R, Purschwitz J, Veith D, Frankenberg-Dinkel N, et al The *Aspergillus nidulans* phytochrome FphA represses sexual development in red light. Current biology: CB. 2005;15(20):1833–8. . 16243030 10.1016/j.cub.2005.08.061

[65] Hoffmann B, Wanke C, Lapaglia SK, Braus GH. c-Jun and RACK1 homologues regulate a control point for sexual development in *Aspergillus nidulans* . Molecular microbiology. 2000;37(1):28–41. . 10931303 10.1046/j.1365-2958.2000.01954.x

[66] Han KH, Seo JA, Yu JH. A putative G protein-coupled receptor negatively controls sexual development in *Aspergillus nidulans* . Molecular microbiology. 2004;51(5):1333–45. 10.1111/j.1365-2958.2003.03940.x . 14982628

[67] Tsitsigiannis DI, Kowieski TM, Zarnowski R, Keller NP. Three putative oxylipin biosynthetic genes integrate sexual and asexual development in *Aspergillus nidulans* . Microbiology. 2005;151(Pt 6):1809–21. Epub 2005/06/09. . 15941990 10.1099/mic.0.27880-0

[68] Ahmed YL, Gerke J, Park HS, Bayram O, Neumann P, Ni M, et al The velvet family of fungal regulators contains a DNA-binding domain structurally similar to NF-kappaB. PLoS Biol. 2013;11(12):e1001750 Epub 2014/01/07. 10.1371/journal.pbio.1001750 24391470 PMC3876986

[69] Mirzadi Gohari A, Mehrabi R, Robert O, Ince IA, Boeren S, Schuster M, et al Molecular characterization and functional analyses of ZtWor1, a transcriptional regulator of the fungal wheat pathogen *Zymoseptoria tritici* . Mol Plant Pathol. 2014;15(4):394–405. Epub 2013/12/18. 10.1111/mpp.12102 . 24341593 PMC6638687

[70] Galagan JE, Calvo SE, Cuomo C, Ma LJ, Wortman JR, Batzoglou S, et al Sequencing of *Aspergillus nidulans* and comparative analysis with *A*. *fumigatus* and *A*. *oryzae* . Nature. 2005;438(7071):1105–15. Epub 2005/12/24. 10.1038/nature04341 . 16372000

