# Supplementary figures and images for: The WOPR Domain Protein OsaA Orchestrates Development in Aspergillus nidulans
Source: PLoS One. 2015 Sep 11;10(9):e0137554. doi: 10.1371/journal.pone.0137554 (PMC4567300; doi:10.1371/journal.pone.0137554)

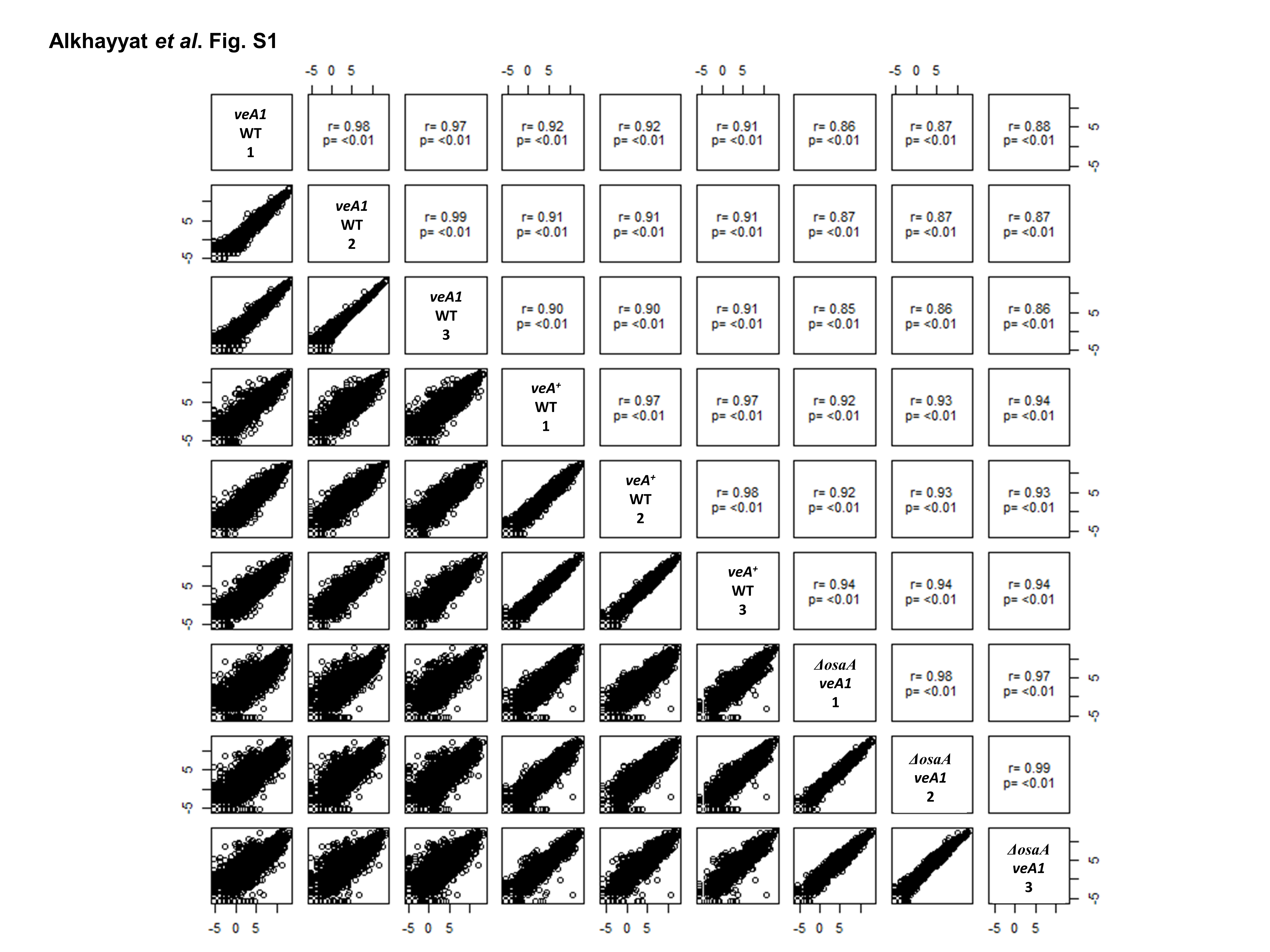

Supplement: S1 Fig — Scattered plot showing the correlation levels among triplicates of each sample. The correlation coefficient R for ΔosaA veA1 was > 0.97, veA1 WT was > 0.97, and veA + WT was > 0.97, all with p-value less than 0.01, indicating the high quality of the RNA-Seq data sets. (TIF) [file pone.0137554.s001.TIF]

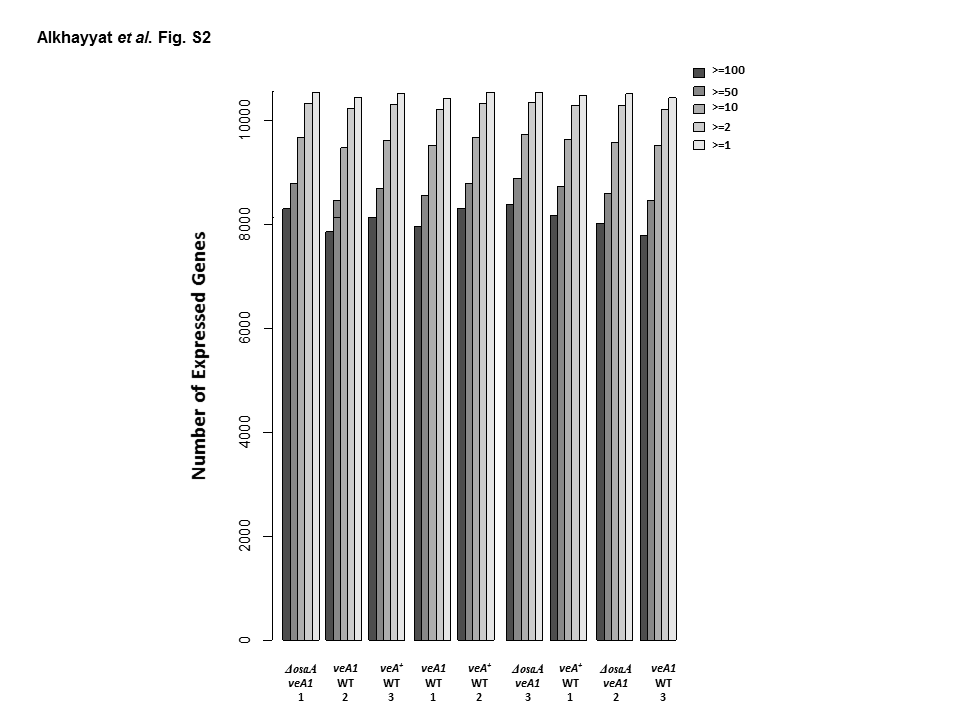

Supplement: S2 Fig — FPKM obtained for ΔosaA veA1, veA1 WT, and veA + WT strains are mapped to 10,536, 10,428 and 10,514 genes, respectively, representing 96.3%, 95.3% and 96% coverage of a total of 10,943 genes predicted by AspGD. (TIF) [file pone.0137554.s002.TIF]

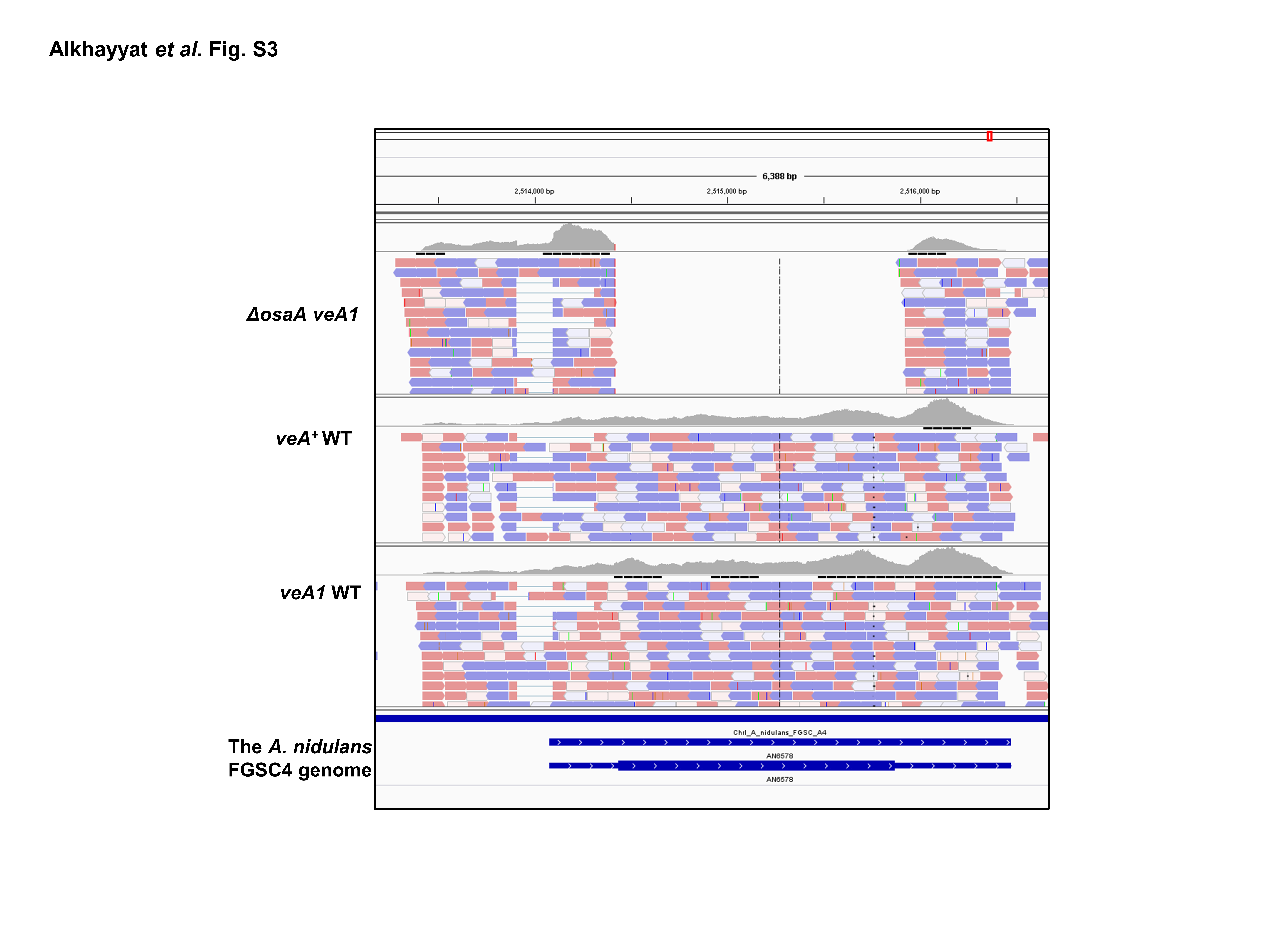

Supplement: S3 Fig — A snapshot from Integrative Genomics Viewer (IGV) software showing the osaA locus (AN6578) in ΔosaA veA1, veA1 WT, and veA + WT strains. (TIF) [file pone.0137554.s003.TIF]

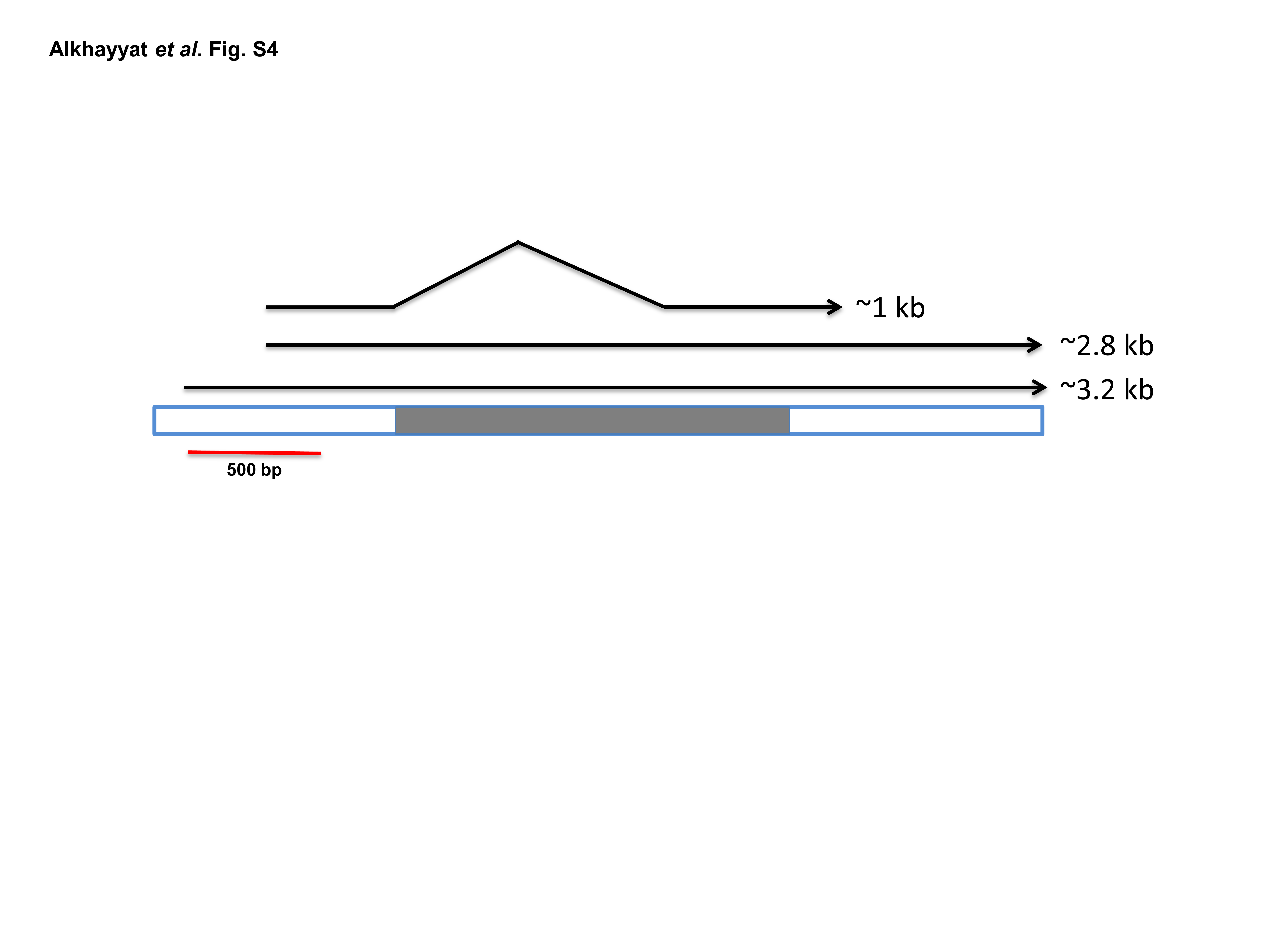

Supplement: S4 Fig — Open box represents osaA locus; grey box represents ORF; arrow line represents transcript; open triangle in transcript ~1kb indicates alternative splicing. (TIF) [file pone.0137554.s004.TIF]

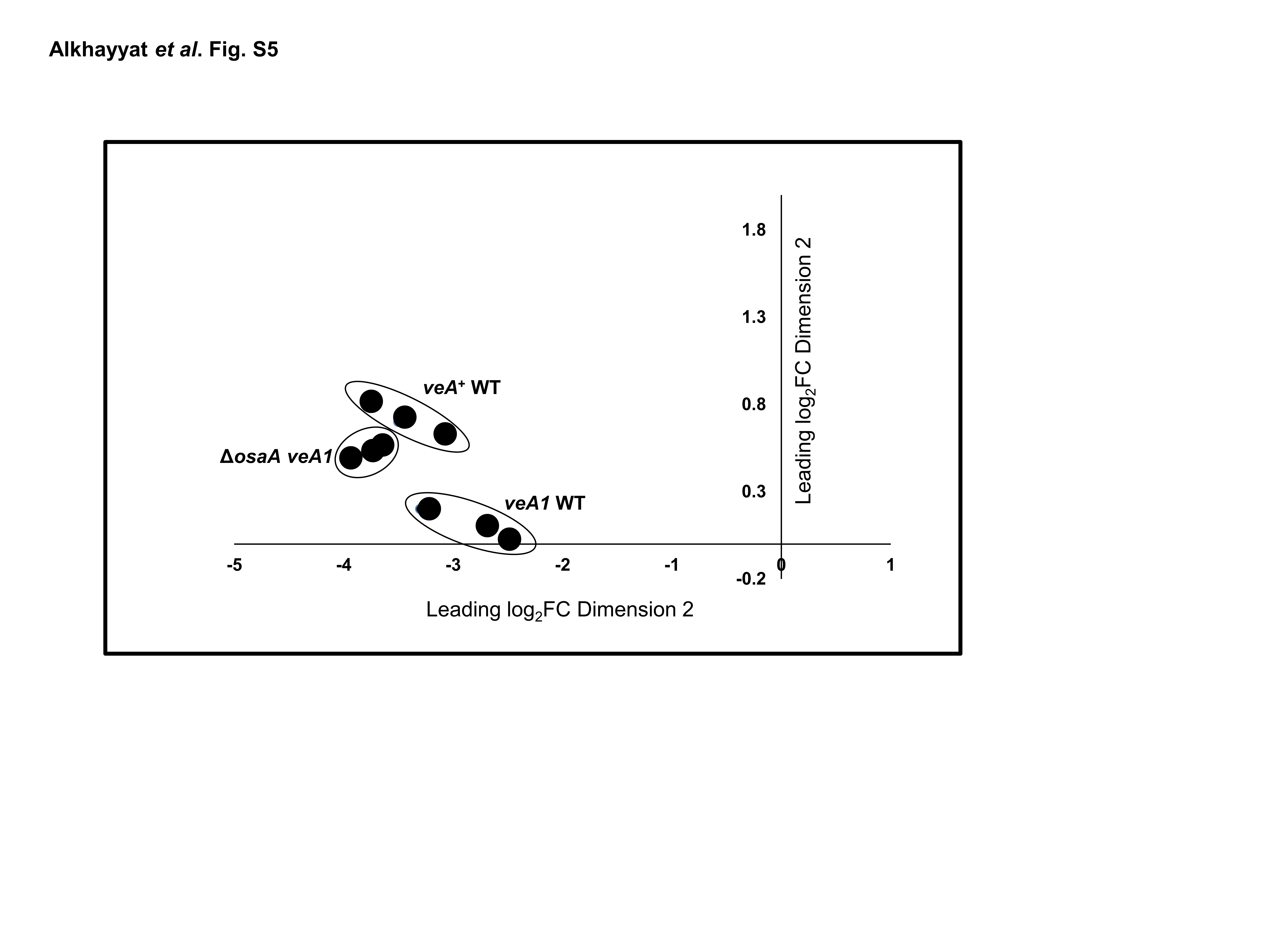

Supplement: S5 Fig — The overall transcriptomic profiles of ΔosaA veA1, veA1 WT, and veA + WT strains examined by a two-dimensional plot. Black circles indicate replicates. (TIF) [file pone.0137554.s005.TIF]
