# Supplementary material for: The WOPR Domain Protein OsaA Orchestrates Development in Aspergillus nidulans
Source: PLoS One. 2015 Sep 11;10(9):e0137554. doi: 10.1371/journal.pone.0137554 (PMC4567300; doi:10.1371/journal.pone.0137554)
Supplement: S1 Table — (DOCX) [file pone.0137554.s006.docx]

**S1 Table.** A list of differentially expressed development-related genes in both *veA^+^* WT and Δ*osaA veA1* relative to *veA1* WT.

| **Gene** | **Log_2_FC** | **FDR** | **Log_2_FC** | **FDR** |
| --- | --- | --- | --- | --- |
|  | *veA+* WT vs *veA1* WT | *veA+* WT vs *veA1* WT | Δ*osaA veA1* vs *veA1* WT | Δ*osaA* *veA1* vs *veA1* WT |
| *MAT1* | 2.743 | 0.002373 | 2.970 | 0.001016 |
| *srrA* | 2.590 | 0.000274 | 2.578 | 0.000157 |
| *preB* | 1.686 | 0.000582 | 2.215 | 0.000115 |
| *veA* | 1.560 | 0.003072 | 1.934 | 0.000341 |
| *tcsB* | 1.574 | 0.001063 | 1.912 | 0.000572 |
| *stuA* | 1.379 | 0.007348 | 1.678 | 0.001256 |
| *swoM* | 0.628 | 0.014521 | 1.441 | 0.000216 |
| *MAT2* | 1.605 | 0.001388 | 1.392 | 0.002 |
| *pbcR* | 1.630 | 0.000582 | 1.320 | 0.002405 |
| *steD* | 0.992 | 0.006023 | 1.278 | 0.004166 |
| *sltA* | *0.058* | *0.058179* | 1.154 | 0.004331 |
| *flbD* | 1.406 | 0.00094 | 1.148 | 0.043363 |
| *ygA* | 0.814 | 0.008816 | 0.993 | 0.001733 |
| *preA* | 1.892 | 0.000422 | 0.986 | 0.004 |
| *slaB* | 0.588 | 0.01336 | 0.985 | 0.002215 |
| *rsmA* | 0.499 | 0.026992 | 0.975 | 0.001999 |
| *myoA* | *0.091* | *0.090722* | 0.966 | 0.004494 |
| *lreA* | 1.125 | 0.003107 | 0.938 | 0.002852 |
| *lreB* | 0.888 | 0.006043 | 0.928 | 0.002745 |
| *mid1* | 0.724 | 0.004924 | 0.880 | 0.003848 |
| *atg1* | *0.061* | *0.061415* | 0.820 | 0.007267 |
| *ugtA* | 1.093 | 0.005179 | 0.797 | 0.002453 |
| *steA* | 0.890 | 0.024111 | 0.791 | 0.011157 |
| *sgdB* | 0.702 | 0.027225 | 0.785 | 0.006494 |
| *phoA* | 0.942 | 0.001766 | 0.761 | 0.002082 |
| *crzA* | 0.770 | 0.023692 | 0.757 | 0.044744 |
| *fadA* | 0.625 | 0.027135 | 0.730 | 0.00934 |
| *flbE* | 0.950 | 0.005637 | 0.622 | 0.013058 |
| *ste7* | 0.621 | 0.037787 | 0.621 | 0.049376 |
| *sskA* | 0.498 | 0.04747 | 0.603 | 0.00618 |
| *steC* | 0.771 | 0.005637 | 0.591 | 0.028871 |
| *csnC* | 0.902 | 0.001998 | 0.583 | 0.006184 |
| *sgdA* | 1.112 | 0.004865 | 0.562 | 0.013987 |
| *nosA* | 1.015 | 0.004704 | 0.558 | 0.04798 |
| *cch1* | 0.643 | 0.007235 | 0.554 | 0.013105 |
| *rhbA* | 1.165 | 0.001422 | 0.532 | 0.01655 |
| *mdmB* | 0.644 | 0.027877 | 0.524 | 0.033765 |
| *tcsA* | 0.708 | 0.007871 | 0.502 | 0.010602 |
| *chsB* | 0.681 | 0.018812 | *0.069* | *0.069431* |
| *bemA* | 0.627 | 0.019524 | 0.410 | 0.046452 |
| *mpkB* | *0.077* | *0.077006* | -0.417 | 0.042172 |
| *fphA* | *0.056* | *0.056321* | -0.434 | 0.028915 |
| *pkaR* | -0.558 | 0.011474 | -0.471 | 0.015722 |
| *yA* | *0.067* | *0.066811* | -0.585 | 0.033539 |
| *sgdE* | *0.085* | *0.085074* | -0.607 | 0.010602 |
| *sidB* | -0.723 | 0.005096 | -0.609 | 0.005583 |
| *orlA* | -0.899 | 0.017843 | *0.073* | *0.073184* |
| *plkA* | -0.499 | 0.030285 | -0.814 | 0.009406 |
| *aroC* | -1.205 | 0.00627 | -0.839 | 0.034272 |
| *ppoC* | -0.967 | 0.004508 | -0.872 | 0.003807 |
| *rodA* | -0.748 | 0.025163 | -0.914 | 0.018178 |
| *chsA* | -0.525 | 0.022621 | -0.978 | 0.001253 |
| *esdC* | *0.064* | *0.063505* | -1.062 | 0.001406 |
| *myoB* | -0.595 | 0.0127 | -1.066 | 0.000506 |
| *prpA* | -1.204 | 0.014436 | -1.101 | 0.02776 |
| *schA* | -1.197 | 0.006224 | -1.135 | 0.001351 |
| *axl2* | -1.645 | 0.001599 | -1.168 | 0.004356 |
| *lsdA* | -0.771 | 0.021465 | -1.170 | 0.008694 |
| *wetA* | -0.910 | 0.008047 | -1.189 | 0.002741 |
| *chpA* | -0.934 | 0.013611 | -1.353 | 0.003108 |
| *rho4* | -0.973 | 0.001597 | -1.395 | 0.00019 |
| *ivoA* | -1.738 | 0.000497 | -1.636 | 9.76E-05 |
| *dewA* | -0.863 | 0.018312 | -1.711 | 0.001889 |
| *gmcA* | -2.741 | 0.00032 | -2.021 | 0.000663 |
| *flbC* | -1.116 | 0.00631 | -2.031 | 0.000111 |
| *gprD* | -2.683 | 0.000515 | -3.084 | 0.000203 |

*****Differentially expressed genes that show p-value and FDR above or equal to 0.05 (insignificant) are in italic.
