# Supplementary material for: The WOPR Domain Protein OsaA Orchestrates Development in Aspergillus nidulans
Source: PLoS One. 2015 Sep 11;10(9):e0137554. doi: 10.1371/journal.pone.0137554 (PMC4567300; doi:10.1371/journal.pone.0137554)
Supplement: S2 Table — (DOCX) [file pone.0137554.s007.docx]

**S2 Table.** Oligonucleotides used in this study.

| **Oligo** | **Sequence** | **Purpose** |
| --- | --- | --- |
| OKH60 | gactctataccaccgtacgccgatat | 5' *argB* |
| OMN33 | AAATAAGCTTGCATGCGC | pRG3-AMA1 sequencing 5′ |
| OMN35 | GCCAGTGAATTCGAGCTC | pRG3-AMA1 sequencing 3′ |
| OKH67 | gtgttaggcctggatcta | 3' *argB* |
| OMN40 | tatttcctcacttagctggggc | Δ*osaA* 5' forward |
| OMN41 | cacaccaactccagtacgtaac | Δ*osaA* 3' reverse |
| OMN42 | caaatgaggcctctaaactggtcatcccctcgaactcttattgtg | Δ*osaA* 5' reverse with 5' *argB* tail |
| OMN43 | caaggtagatccaggcctaacaatttgcgactcttttcttgccg | Δ*osaA* 3' forward with 3' *argB* tail |
| OMN44 | ctggatgtcttgttgactctgc | Δ*osaA* 5' nest |
| OMN60 | cgcaaggagtcaggtaactaag | Δ*osaA* 3' nest |
| oFA27 | TTCCTGACGAAAGACCTGGG | *AflwprA* 5` forward |
| oFA29 | GCGCTTCCATATACACCAGA | *AflwprA* 3` reverse |
| oFA31 | AATGGTCAACGGCACCACCA | *AflwprA* ORF 5' |
| oFA32 | ATACCTGCGGTTGAGCTGGA | *AflwprA* ORF 3' |
| oHS168 | CAGTTGTCCGAGTTTGTGCTGGAG | 5' *biA* |
| oHS169 | ATCTGCGGGAGACTCACTAAGAGCC | 3' *biA* |
| OJA142 | CTGGCAGGTGAACAAGTC | 5’ *brlA* probe |
| OJA143 | AGAAGTTAACACCGTAGA | 3’ *brlA* probe |
| OJA154 | AGCTCTTCAGAATACGTC | 5’ *abaA* probe |
| OJA155 | GTTGTGAGATGCCTCCAT | 3’ *abaA* probe |
| OMN66 | TTTCCAGATCCTTCGCAG | 5’ *vosA* probe |
| OMN63 | ATAGAAACAGCCACCCAG | 3’ *vosA* probe |
| oKH181 | ggctgtagtcgctttgtt | 5' *veA* probe |
| oKH182 | gcccagtgtaagaaagga | 3' *veA* probe |
| oMN340 | atgggatcactagaggctggac | 5' *nsdD* probe |
| oMN341 | ttaatgactcctcggtgacacc | 3' *nsdD* probe |
| oHS490 | ATGCCGGCAGCACCGAGAAAG | 5' *nosA* probe |
| oHS491 | TCAAAGAAGAAGGTAGTTCCAACCG | 3' *nosA* probe |
